# Graph Convolutional Neural Network-Enabled Frontier Molecular Orbital Prediction: A Case Study with Neurotransmitters and Antidepressants

**DOI:** 10.1021/acs.jcim.5c00724

**Published:** 2025-07-17

**Authors:** Rivaaj Monsia, Stewart C. Gundry, Molly L. Mohr, Macey A. Smith, Sudeep Bhattacharyya, Sanchita Hati

**Affiliations:** Department of Chemistry and Biochemistry, 14747 University of Wisconsin—Eau Claire, Eau Claire, Wisconsin 54702, United States

## Abstract

With the advancement of artificial intelligence-embedded methodologies, their application to predict fundamental molecular properties has become increasingly prevalent. In this study, a graph convolutional neural network fingerprint-enabled artificial neural network (GCN-ANN) was utilized to probe the relationship between the chemical hardness of neurochemicals and their affinities for neuroreceptors. The GCN-ANN model was derived using a training set of B3LYP-calculated HOMO and LUMO energies of >110,000 molecules. A benchmark study of 45 neurochemicals produced consistent hardness and electronegativity values across the three density functionals, namely, B3LYP, ωB97XD, and M06-2X. However, the computed energetics varied significantly when the Hartree–Fock theory was used. The scrutiny of binding affinities, hardness, and GCN-ANN-derived substructures of neurochemicals reinforces the notion that human brain receptors interact with neurochemicals based on Pearson’s Hard–Soft Acid–Base (HSAB) principle. In summary, this machine-learning-embedded study offers physical insights into the interactions between neurochemicals and neuroreceptors, which could lead to the development of more targeted and effective antidepressants, thereby addressing anxiety and depression with greater precision and immediacy.

## Graphical abstract

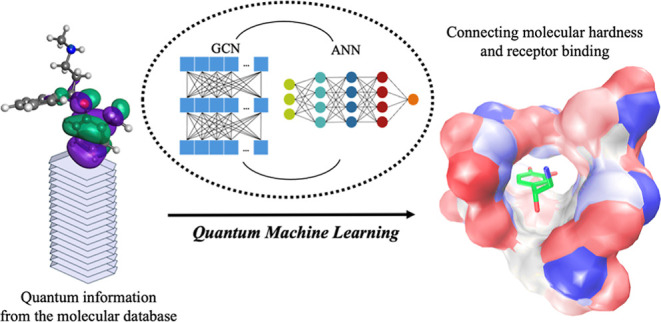

## Introduction

Significant advancements have recently been made in applying deep learning to molecular design and predicting molecular structures and properties.^1–14^ The reason is largely due to the enhanced computational efficiency of the machine learning (ML) algorithms compared to quantum chemical methods, which require numerical solutions to the multielectronic Schrödinger equation.^2,6,14^ In recent years, numerous studies have reported the prediction of molecular properties using ML, which relies on data-based modeling instead of physics-based rules.^5,8,10,14–16^ Using significantly large molecular databases, these algorithms have used pattern recognition to predict quantized molecular properties, resulting in quantum ML.^4,10,15,17,18^ However, only a few of these studies focused on explaining the physical significance of artificial intelligence-derived models as they are applied to chemical systems. One such actively researched area involves the prediction of frontier molecular orbitals of small molecules using ML algorithms, especially with graph-based neural networks.^1,2,4,19–22^ However, the implication of these models in pinpointing the frontier molecular orbitals that can explain molecular properties such as polarity and intermolecular interactions has remained underexplored.

Deep learning models based on the graph convolutional neural network (GCN) have been successful in identifying molecular substructures linked to certain molecular properties, such as solubility, affinity for a specific target, etc.^1,9,11^ In an earlier study, a trainable GCN fingerprints-enabled artificial neural network (GCN-ANN) protocol was reported,^23^ which is capable of extracting the substructure of hit molecules for a specific target protein. In the present study, the GCN-ANN-based learning algorithm has been extended to examine the interplay of frontier orbitals energies, chemical hardness, and intermolecular interactions, especially in the context of interactions of neurotransmitters and antidepressants with neurotransmitter receptors.

Mental health is a worldwide problem with approximately 280 million people suffering from depression according to the World Health Organization report released in March 2023.^24–26^ Globally, an estimated 4.4% and 3.6% of the population suffer from depression and anxiety disorders, respectively, both of which are linked to the imbalance of neurotransmitters.^25–31^ Antidepressants are prescribed to countless individuals worldwide to treat anxiety and depression. However, existing FDA-approved antidepressants often have limitations, including resistance in some patients and undesirable side effects, there’s a growing need for antidepressants that target receptors more precisely and with fewer side effects.^26,29–34^ To develop more effective antidepressant drugs with minimal side effects, it is important to understand the chemical properties of neurotransmitters and antidepressants and how they interact with various neuroreceptors.

The electron donor–acceptor properties of neurotransmitters are known to be closely associated with their agonistic and antagonistic behaviors during receptor binding.^35^ Structure–activity relationship based on the chemical hardness of neurotransmitters has also been reported.^36^ Thus, new approaches to fine-tune these molecular properties hold great potential to create antidepressants targeting a specific neurotransmitter, which could lead to improved therapeutic outcomes. A rational approach to drug design involves analyzing the molecular hardness of compounds with strong binding affinities for target proteins and identifying the specific substructures that are preferred by the active site. Herein, a case study that explored the GCN-ANN method for computing the molecular properties such as electronegativity and hardness of neurotransmitters and common antidepressant drugs (Table 1) is reported. Furthermore, a molecular docking study was performed to explore Pearson’s Hard Soft Acid Base (HSAB)^37^ principle and understand the interactions between neurochemicals and neuroreceptors. The GCN-ANN-predicted substructures of neurochemicals and their corresponding binding energetics provide a framework for successful antidepressant design.

**Table 1:** Neurotransmitters and Antidepressants Used in This Study

neurotransmitters compound name	antidepressants compound name (brand name)
1. choline	29. citalopram (Celexa)
2. chlorpromazine	30. sertraline (Zoloft)
3. barbital	31. paroxetine (Paxil)
4. aspartic acid	32. escitalopram (Lexapro)
5 amphetamine	33. atomoxetine (Strattera)
6. adrenaline	34. desvenlafaxine (Pristiq)
7. acetylcholine	35. duloxetine (Cymbalta)
8. tyramine	36. levomilnacipran (Fetzima)
9. taurine	37. tramadol (Ultram)
10. serotonin	38. venlafaxine (Effexor)
11. phenethylamine	39. amitriptyline (Elavil)
12. noradrenaline	40. amoxapine (Asendin)
13. *m*-tyramine	41. desipramine (Norpramin)
14. haloperidol	42. doxepin (Silenor)
15. glutamic acid	43. bupropion (Wellbutrin)
16. dopamine	44. vortioxetine (Trintellix)
17. diazepam	45. mirtazapine (Remeron)
18. g-aminobutyric acid	
19. taurocyamine	
20. nicotine	
21. muscarine	
22. CH_3_COOCH_2_CH_2_N^+^ (C_2_H_5_)_3_	
23. CH_3_COOCH_2_N^+^(CH_3_)(C_2_H_5_)_2_	
24. CH_3_COOCH_2_CH_2_N^+^(CH_3_)_2_(C_2_H_5_)	
25. impramine	
26. oxotremorine	
27. fluoxetine	
28. milnacipran	

## Theory and Methods

Electronic structure calculations were performed on the hybrid GPU-CPU BOSE supercomputer with 61 nodes and 3904 cores, located at the Blugold Center for High-Performance Computing, University of Wisconsin-Eau Claire. Each node contains two CPUs, equipped with 2.3 GHz/32-core AMD EPYC 7452, and is connected through Hewlett-Packard Slingshot internode connection. GPU nodes equipped with NVIDIA Tesla V100 GPU cards were used for ML calculations. The quantum chemistry package QCHEM^38^ was used for all electronic structure calculations. Visualization and editing of the structure all small molecules were done using IQmol.^39^ Structures of 28 neurotransmitters and 17 antidepressants were built according to their known chemical structures obtained from PubChem (Figure 1).^40^ Coordinates of all protein structures were obtained from the Protein Data Bank^41^ and were visualized using the Visual Molecular Dynamics (VMD) program.^42^ Interactions between the ligand and active site side chains were studied using VMD as well as the LigPlot+ program.^43,44^ The PDB2PQR^45^ program was employed to assign partial atomic charges of protein atoms using CHARMM^46^ forcefields. Electrostatic potentials were developed using these charges, mapped onto the surface of active site residues, and visualized using VMD. Machine-readable molecular substructures were read and written using RDKit.^47^ Scikit-learn (version 1.4) library^48^ was used to compute the precision, recall, and receiver operating characteristics (ROC). The technical details of neural network architecture were implemented in PyTorch.^49^

**Figure 1:**
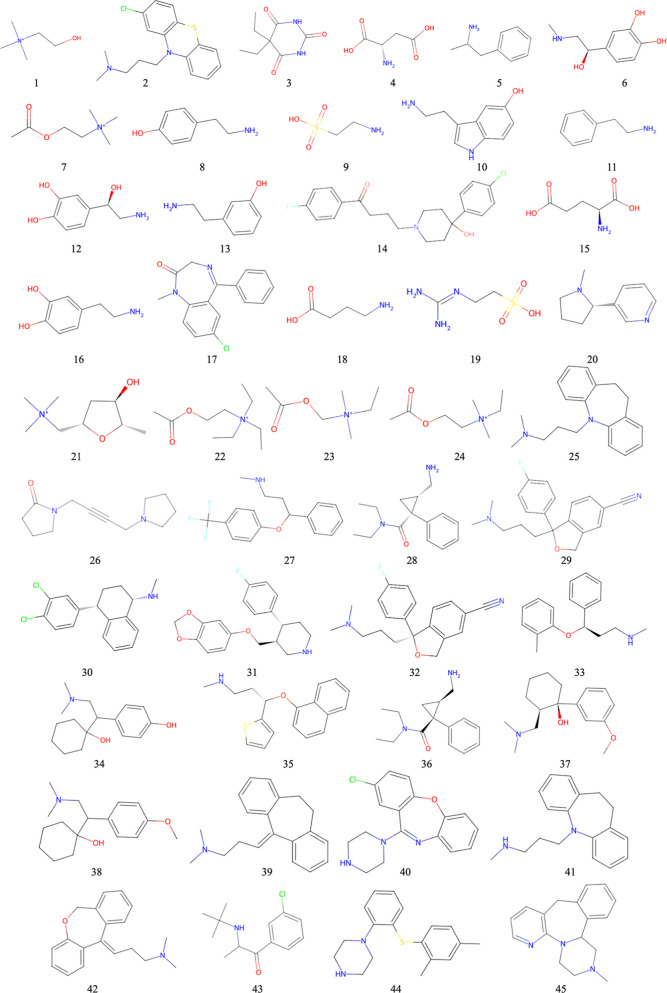
Two-dimensional chemical structures with spatial arrangements of atoms for molecules 1–45 (28 neurotransmitters and 17 antidepressants, as in Table 1) used in the present study.

### Electronegativity and Hardness of Molecules

Electronegativity and hardness are important chemical properties that characterize a molecule. Electronegativity (χ) is a measure of the tendency of a species to attract the electron density,^50,51^ which is equal to the negative of the electronic chemical potential, μ.^51^ The higher the electronegativity of an atom in a molecule, the stronger its attraction for the bonding pair of electrons. On the other hand, hardness (η) in a chemical species is defined as a measure of the resistance to deformation of its electron density.^52^ In other words, η measures the tendency of a chemical species (atom, ion, or molecule) to resist the polarization of its electron cloud.^51^ Chemically hard compounds are less polarizable, and chemically soft ones are more polarizable.

The hardness η is an important chemical property that provides information about molecular reactivity and selectivity.^53–56^

χ and η of a chemical species can be calculated from the energies of the highest occupied molecular orbital (HOMO) and the lowest unoccupied molecular orbital (LUMO) following eqs 1 and 2, respectively.^57^

1 $$\chi =-\frac{1}{2}\left(\varepsilon_{HOMO}+\varepsilon_{LUMO}\right)=-\mu$$

2 $$\eta =-\frac{1}{2}\left(\varepsilon_{HOMO}-\varepsilon_{LUMO}\right)$$

According to Koopman’s theorem,^58^ under the Hartree–Fock assumption, the HOMO and LUMO energies of a closed shell species match the ionization potential (IP) and electron affinity (EA), respectively. For the Hartree–Fock calculations, the geometry optimization yielded the minimum energy conformation of a molecule, and the orbital energies are obtained as the eigenvalues of the diagonalized Fock matrix. It has been theorized that in density functional treatment of electron correlations, Janak’s derivation produces an analogous relationship between molecular orbitals and the electron transfer energetics.^59^ For a molecule, X, computations required gas-phase geometry optimization of the neutral (X), anionic (X^–^), and cationic (X^+^) species.^60^ The ionization potential (IP) is obtained from the difference of energy of neutral and cationic species as follows

3 $$IP=E\left(X^{+}\right)-E\left(X\right)$$

In the above equation, *E*(X^+^) and *E*(X) represent the Born–Oppenheimer potential energies of the geometrically optimized cationic and neutral species, respectively. A similar expression was used to compute electron affinity (EA) using the Born–Oppenheimer potential energy *E*(X^–^) of the anionic species

$$EA=E\left(X\right)-E\left(X^{-}\right)$$ 4

Following Parr’s derivation,^50^ χ and η are expressed in terms of IP and EA as follows

5 $$\chi =\frac{\left(IP+EA\right)}{2}$$

6 $$\eta =\frac{\left(IP-EA\right)}{2}$$

### Electronic Structure Calculation

The electronic structure of each molecule was determined either using the Hartree–Fock (HF) method or the Kohn–Sham Density Functional Theory (DFT).^61^ A basis set of 6-31G­(d,p) was used for all HF calculations, while DFT calculations for B3LYP,^62–65^ M06-2X,^66,67^ and ωB97XD^68^ were carried out using a 6-311++G­(d,p) basis set.^69^ In the present study, χ and η were calculated for 28 neurotransmitters^36^ and 17 common antidepressants. The geometry-optimized structure of each molecule and its various charged state were used to determine the highest occupied molecular orbital (HOMO) and lowest unoccupied molecular orbital (LUMO) energies. Additionally, the IPs and EAs were computed using eqs 3 and 4 and were utilized to calculate χ and η (eqs 5 and 6) for the three above-mentioned DFT functionals.

### Graph Convolutional Neural Network-Based HOMO–LUMO Prediction

A GCN that produces real-valued, differentiable substructure-based fingerprints was used in tandem with an ANN to predict HOMO and LUMO energies. This stitched GCN-ANN protocol consists of an end-to-end architecture to generate a differentiable, real-valued molecular fingerprint, *f*, from a molecule’s SMILE representation (Figure 2). This is an extension of the work of Duvenaud et al.,^11^ where trainable substructure-based fingerprints that are task-optimized, in this case HOMO/LUMO energies were generated. Instead of using ubiquitous molecular fingerprints like Morgan fingerprints^71^ and/or extended-connectivity fingerprints (ECFPs),^72^ this method utilizes neural network-based operations with trainable parameters to undergo graph-based convolutions on the molecular graph (Figure 2), in essence pooling information from neighboring bonds and atoms to update the features/information on each atom in a molecule. Hence, this method is substructure-based due to these graph-based operations, which, through iterative steps, produces the final fingerprint, *f*. The differentiable fingerprint, *f*, is then mapped to the input layer of an ANN (Figure 2). The ANN has a hyperparameter-defined number of hidden layers with rectified linear unit (ReLU) nonlinearities as activation functions. The output of the ANN is a continuous value representing the predicted HOMO or LUMO energy.

**Figure 2:**
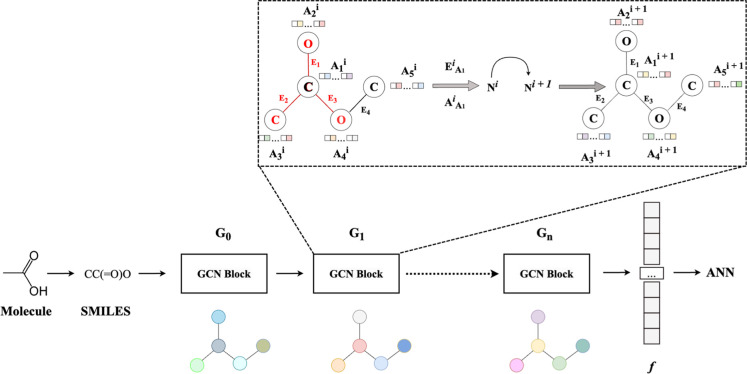
Operations utilized to update a single atom’s feature matrix at degree-step N^*i*^ using acetic acid and degree-step *i* = 1 as an example. The inset illustrates the steps within a representative GCN block: the edge (E_1_, E_2_, E_3_) and atom (A_2_, A_3_, A_4_) features of all atoms adjacent to the central carbonyl carbon (C) are highlighted in red and pooled to update the central carbonyl carbon’s feature matrix. The degree (*i*) defines the subset of atoms (A^*i*^) and edges (E^*i*^) comprising the *i*-hop neighborhood of the atom, and the arrows indicate the direction of information passed during the operation. The pooled E^*i*^ and A^*i*^ matrices are then used to update the carbonyl carbon’s node feature matrix, N^*i*^ to N^*i*+1^, and this process is repeated for every atom for every possible degree. The final fingerprint obtained through a series of GCN blocks, *f*, is then fed to an ANN input layer.

A public database containing 111,725 molecules available via a figshare repository^73^ was used for the current study. The retrieval of these molecules while creating the database was reported earlier by Pereira et al.^3^ The electronic structure calculations of these molecules were carried out at the level of Kohn–Sham density functional theory (DFT)^61^ using B3LYP functional and 6-31 G­(d) basis set. The molecular orbital energies and corresponding structures were used to train, test, and validate HOMO and LUMO-focused models. The data set was randomly partitioned into 70–15–15 percentage splits for training, testing, and validation data sets, respectively. Thus, 77,808 molecules were used in the training data set, while each of the testing and validation data sets contained 16,673 molecules. Ten models with varying hyperparameters and architectural parameters were independently trained for a maximum of 200 epochs. Hyperparameters were generated by varying both GCN and ANN architectural parameters and included fingerprint sizes, learning rates, batch size, and L2 regularization. Optimal values for L2 regularization and architecture are instrumental in combatting the overfitting of data. Preliminary experiments showed no significant improvement using dropout layers, so they were not included in the final model architecture. The loss function simply calculated the mean squared error between predicted and the true values in a specific set. Lastly, early stopping was enabled if and only if validation losses were within a range of 0.01 through 20 sequential epochs (Table 2).

**Table 2:** Details of the GCN-ANN Hyperparameters Used in This Study

	GCN convolutional layers	ANN layers	batch size	learning rate	L2 regularization rate	fingerprint length
hyperparameter set	3, 4	2, 3, 4	128, 256	0.001, 0.0003, 0.0001	0, 0.0001	32, 64, 128

These models were then assembled into ensembles and trained for a few epochs. Compared to evaluation with a single model, the use of an ensemble refers to the combination of several unique models into a single prediction. A property of a good ensemble is the diversity of its constituent models. A higher diversity of models results in an aggregation of unique and comprehensive feature recognition and correlations that, theoretically, result in better predictions. In this study, unique sets of hyperparameters and architectural parameters were used to define and train each model. After training each model separately, the ensemble is achieved by connecting the outputs using a simple, minimally trained linear layer with a corresponding activation function.

### Evaluation Metrics

To evaluate the HOMO and LUMO models, four metrics are reported for each model, the root-mean-square error (RMSE), the mean absolute error (MAE), the coefficient of determination *R* ^2^, and the symmetric mean absolute percentage error (SMAPE). RMSE measures the average deviation between the predicted and actual values of a regression model, while MAE, computed from the average of the absolute difference between individual pairs of predicted and actual values in the data set, thus, providing an insight into a regression model’s accuracy. The coefficient of determination, *R* ^2^ relays the goodness of fit of a statistical model, measuring the proportion of variation between the predicted values and actual values of a data set. SMAPE is defined by eq 7

7 $$SMAPE=\frac{100}{N}\sum_{i=1}^{N}\frac{\left|y_{i}-x_{i}\right|}{\left(\left|y_{i}\right|+\left|x_{i}\right|\right)}$$

where *x* and *y* are the actual and predicted variables, respectively, and *i* indicates the number of data points ranging from 1 to *N*. SMAPE is another accuracy-based metric, similar to MSE and RMSE, and measures the average absolute error in percentage units between the actual and predicted values of a data set. The SMAPE value ranges between 0 and 100%.

### Fingerprint Analysis for Substructure Identification

A benefit of using the GCN-ANN method described above is the generation of an interpretable fingerprint. The process of substructure extraction using maximal fingerprint activation represents the model’s ability to interpret the importance of substructures at a human-comprehensible level based on common knowledge of functional groups. Since the graph-based pooling operation of the GCN-ANN provides information about atomic neighborhoods, the substructure information essentially guides the model’s learning. The correlation between specific chemical properties and substructures may be extracted by computing the maximal activation of a fingerprint—the one-dimensional vector *f*, at each of its indices *i*, corresponding to a unique, learned molecular feature. The maximal activation for the *i*th index of the feature vector, or *f*_*i*_, is the largest value generated by the model across a set of molecules. As described earlier, the feature vector linked to each node is updated by pooling the aggregated information containing the feature vectors of its neighbors for each degree as defined in Figure 2. Thus, the substructure associated with the maximal activation at index *i* can be determined by retrieving the *k*-hop neighborhood of the most activating atom in the molecule after pooling information at degree-step *k*. We define *k* as the possible radii of a substructure, ranging from 1 to the maximum radius, which in this study is set to four. Substructure radii greater than four have been shown to provide negligible performance improvements while increasing redundancy and reducing computational efficiency.^11^

Methodologically, to extract the significant substructures associated with a given task, the pooling operation is performed for all molecular substructures in a given set of molecules. In turn, the contribution of each substructure is ascertained within a certain index by noting the highest numerical contribution of the corresponding fingerprint index. Note that the specific index is arbitrary, and maximally activating substructures are retrieved for all indices in the fingerprint.

### Computational Docking of Neurotransmitters and Antidepressants into Target Receptors

A docking study was conducted using AutoDockFR^74^ to assess the binding energies of neurotransmitters and antidepressants for serotonin and noradrenaline receptors, as commonly prescribed antidepressants are targeted toward these two receptors.^34^ The binding energies for the receptor–neurochemical complex were analyzed in the context of the HSAB theory according to which the hard neurochemicals will bind more tightly to hard receptors, and conversely, soft molecules will bind more tightly to soft receptors. The X-ray crystal structure of the serotonin 1A or 5-HT1A receptor (PDB code: 7e2y) and noradrenaline transporter or NAT (PDB code: 8wtv) were obtained from the Protein Data Bank.^41^ Additionally, muscarinic acetylcholine or M3R receptor (PDB code: 4u14) and γ-aminobutyric acid or GABA_A_ receptor (PDB code: 8g5f) were included to test the HSAB principle for neurotransmitter–receptor interactions.

## Results and Discussion

The computed values of χ (eq 5) and η (eq 6) for 28 neurotransmitters and 17 antidepressants are provided in Tables S1 and S2. The 6-311++G­(d,p) basis set was chosen based on its demonstrated reliability for electronic structure calculations on a broad range of compounds.^76,77^ The inclusion of both diffuse (++) and polarization (d,p) functions is crucial for the accurate modeling of electronic wave functions and valence chemistry. The neurotransmitters’ χ and η values obtained using B3LYP are comparable to published values.^78^ The χ and η values for all molecules were also computed using two other DFT functionals, namely, ωB97XD and M06-2X (Figure 3, Tables S1 and S2). Results from these computations exhibited a trend similar to that of B3LYP. This indicates that the electron correlation effect was modeled effectively. Although, the three correlational functionals were different in their theoretical treatment of electron–electron interactions, a consistent measure of the frontier molecular orbital density distribution is evident in their overlapping trend of the computed energetics for these molecules. A significant increase (>2 eV) in both χ and η was observed for neurotransmitters (1, 7, and 21–24 in Table 1 and Figure 3) containing quaternary ammonium ion, presumably due to the increased nuclear charge resulting in tighter packing and increased electron density. Computed χ in HF calculations, however, was underestimated by about 1 eV (cyan, Figure 3a,b) compared to the DFT calculated values. The same analysis also exhibited an overestimation of the η values (Figure 3c,d) by 2 eV compared to DFT. This difference is likely due to the well-known limitations of the HF theory, which completely ignores electron correlation, and thus resulted in localized density and a larger HOMO – LUMO gap, i.e., hardness.

**Figure 3:**
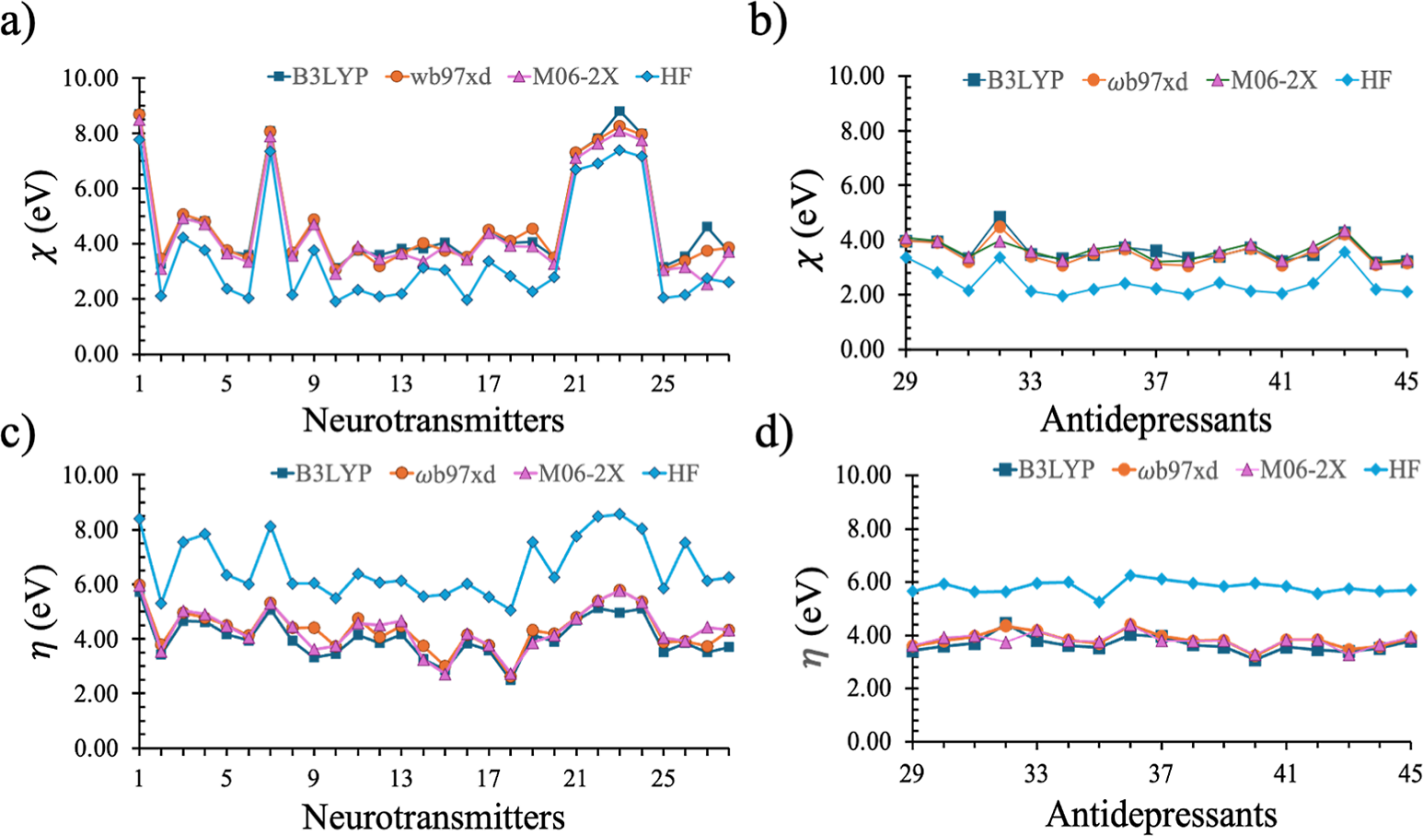
Plots of electronegativity (using eq 5) and hardness (using eq 6) values for 45 neurochemicals (28 neurotransmitters and 17 antidepressants) using different functionals. The numbering scheme is the same as provided in Table 1. The basis set 6-31G­(d,p) was used for HF calculation. DFT calculations with B3LYP, M06-2X, and ωB97XD were performed using the 6-311++G­(d,p) basis set.

For B3LYP functional, the χ and η values were computed using two different theoretical protocols—through explicit determination of IP and EA (eqs 5 and 6) and from HOMO and LUMO energies (eqs 1 and 2). To verify if the energetics are consistent, the root-mean-square error (RMSE) between the comparable χ and η values in the data sets was calculated (Figure S1). The calculated RMSE for χ was ∼0.5 eV (Figure S1a), indicating that χ values in the two data sets are comparable across B3LYP. To examine, if the energetics trend remained consistent across other density functionals, the χ values computed from explicit determination of IP and EA (eq 5) across all DFT functionals were plotted against B3LYP-computed χ using HOMO and LUMO energies (eq 1) (Figure S2a–c). The observed correlation coefficients were >0.95, and the slope was close to unity. Additionally, the HF-derived χ (eq 1) was also compared (Figure S2d), and it exhibited excellent correlation but was underestimated χ by about 1.5 eV compared to B3LYP-computed energies (Figure S2d). Thus, the B3LYP-computed midpoint of the HOMO and LUMO energies provides a reliable estimation of the electronegativity of molecules.

A similar comparative study was also carried out for η using the computed data sets. As illustrated in Figure S1b, there is a consistent underestimation of η values by the protocol using HOMO – LUMO energy than that computed using explicit IP and EA. The computed RMSE between the two methods was 1.3 eV. The comparative analysis was extended to other functionals—B3LYP, ωB97XD, and M06-2X—by plotting the η computed using IP and EA (eq 6) against the same obtained from B3LYP-calculated HOMO and LUMO energies (eq 2) (Figures S1b and S3). A linear trend was obtained from the plots, with the slope ranging from 1.3 to 1.5. The plots also exhibited a few outliers (Figure S3), which were also observed for the HF-computed η values using eq 2. Both these analyses demonstrate that HOMO and LUMO energies are accurate descriptors for computing electronegativities as well as providing a reliable trend of hardness.

### GCN-ANN-Computed Molecular Orbitals Energies

Since HOMO and LUMO energies produced predictable molecular properties, a GCN-ANN model specific to the purpose of molecular orbital energy prediction was developed using B3LYP-calculated HOMO and LUMO energies. The model was blind to the test set (and validation set) and was only trained on the train set where the train, test, and validation sets are partitions (70–15–15) of the original data set. The results of the assessment based on the four metrics described in the Method Section are provided in Table 3. The metrics were calculated with the test set, which was comprised of 15% of the original randomly shuffled B3LYP-calculated HOMO and LUMO energies (eV).

**Table 3:** Assessment of the GCN-ANN Model for the Calculation of HOMO, LUMO, Electronegativity, and Hardness^a^

	RMSE (eV)	MAE (eV)	*R* ^2^	SMAPE (%)
HOMO	0.118	0.087	0.966	0.77
LUMO	0.091	0.065	0.989	4.13
χ	0.074	0.056	0.981	0.79
η	0.075	0.057	0.981	1.51

a All energy quantities are in eV.

From the three metrics of accuracy, RMSE, MAE, and SMAPE (eq 7) in Table 3, it can be concluded that the GCN-ANN model can accurately predict both HOMO and LUMO energies of molecules. Molecules included in the present study are all less than one hundred atoms in composition, so it can efficiently and accurately determine significant substructures that contain the orbitals representing HOMO and LUMO as well as their energies. Although the SMAPE of the LUMO model is relatively higher than that of the HOMO model (Table 3), it still can be considered a reasonably accurate model based on the low RMSE and MAE. Furthermore, the coefficient of determination (*R* ^2^) for both HOMO and LUMO models (Figure 4) describes the goodness of fit between the predicted values and observed values. As indicated by the RMSE, the electronegativity (χ) and hardness (η), computed using the HOMO and LUMO energies, also exhibit reliable assessment metrics (Table 3 and Figure 4). However, of note is the relatively larger SMAPE for LUMO energies (Table 3). This is due to the range of the distribution of LUMO energies in the data set. There are a number of data points close to zero (Figure 4b), and if the true and the predicted values are of opposite signs (<1%), the calculation would produce a % error of exactly 100—the upper bound of SMAPE. The occurrence of large percentage errors from small errors in prediction, when the data values are close to zero, is a known limitation of SMAPE. Nevertheless, the error for LUMO energies is still relatively small and does not impair the overall performance of the model.

**Figure 4:**
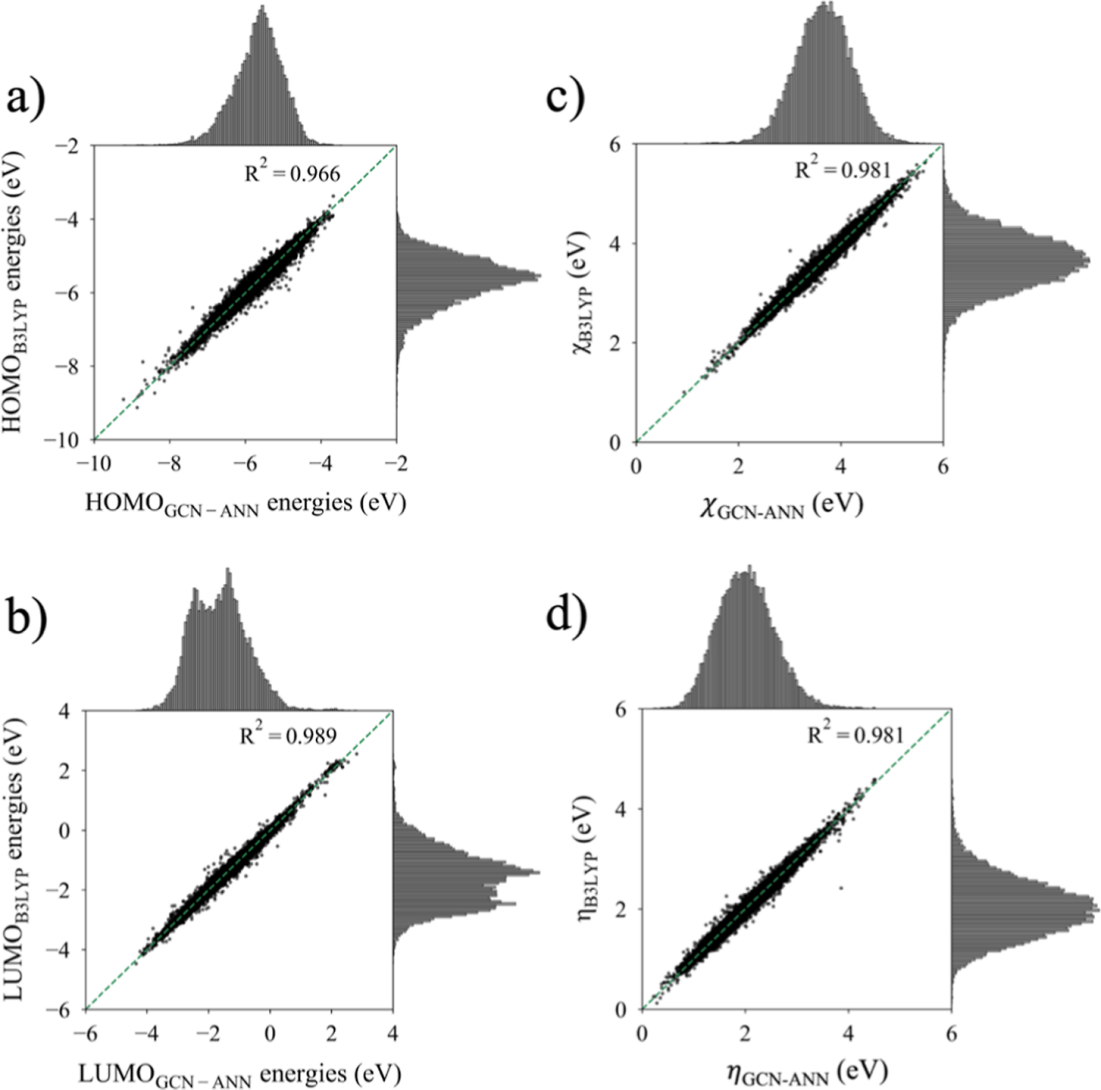
Scatter plots depicting the GCN-ANN-predicted and B3LYP-computed HOMO energies, LUMO energies, the electronegativity (χ) (calculated using eq 1) values, and the hardness values (η) (calculated using eq 2) of the test set. Marginal histograms show GCN-ANN-predicted and B3LYP-computed distributions for each of the aforementioned values.

The reliability of the GCN-ANN architecture is also ascertained from a comparison of the assessment data reported by Pereria et al.^3^ using the same database^73^ utilized in the present study. The study reported HOMO/LUMO energies and their gaps by using Random Forest algorithms,^80^ which were trained on the basis of different molecular descriptors. In particular, authors used a comparable training set of 88,537 molecules (77,808 molecules in the present study) and a smaller test set of 9989 molecules (16,673 molecules in the present study). Compared to their best performing predictors, namely, Md descriptors, Substructure Count, and PubChem fingerprints, *R* ^2^, RMSE, and MAE, the GCN-ANN produced superior results (Table S7).

### Efficient Training Process

To ensemble, ten models were trained for the prediction of HOMO and LUMO energies separately. Figure 5 shows average training time per epoch in seconds and error bars representing standard deviation. All models used for this calculation were batch-trained using a maximum of 250 epochs. Early stopping was utilized during model training once the validation loss converged, improving efficiency and preventing overfitting. Consequently, the majority of the total training time (excluding very little time for validation predictions) can be extrapolated by multiplying the duration of a single epoch by the total number of epochs.

**Figure 5:**
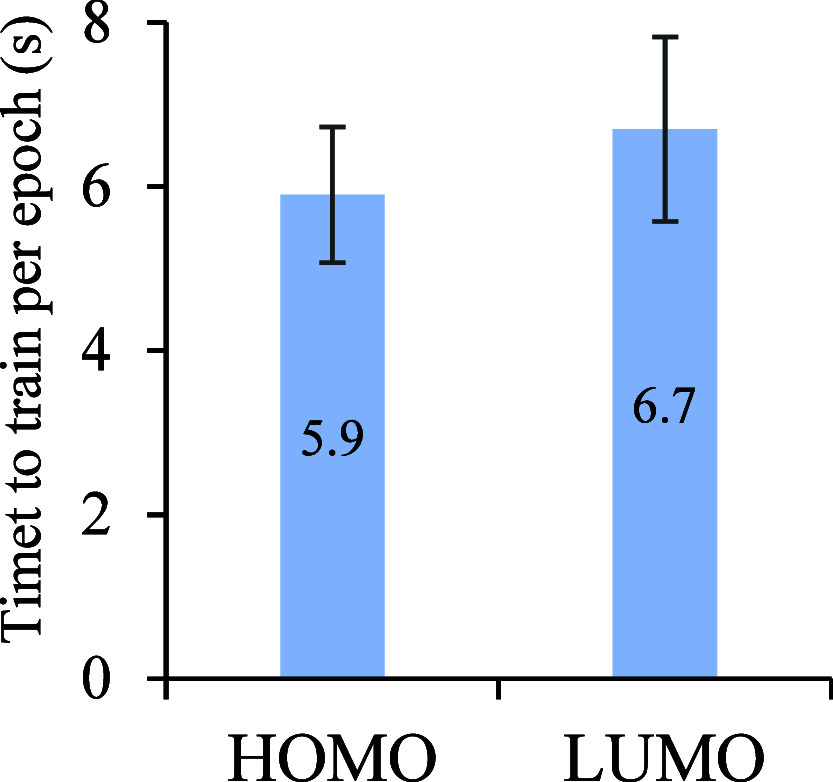
Mean and standard deviation of training per epoch for the HOMO and LUMO models in seconds. The mean and standard deviations were averaged over the epochs of 10 models with batch size 256 and a near-optimal set of hyperparameters.

The results in Figure 5 were averaged across an entire batch of 10 models with batch size 256 and a set of hyper and architectural parameters that were experimentally observed as optimal in training, in terms of performance. Hence, training these models is quite parsimonious and easily achievable given GPU acceleration. Training with larger data sets consisting of millions of molecules also seems feasible even with a single GPU. The training of an ensemble and substructure analysis is quite fast since the ensembling requires only 3–5 epochs to train the linear layer, and substructure analysis only requires predictions on a specific subset of molecules.

### Quality of Hardness Prediction

The calculated HOMO and LUMO energies using the GCN-ANN model were used to assess the electronegativities (eq 1) and hardness (eq 2) of the molecules (Tables S3 and S4). The comparison of the HOMO, LUMO, χ and η by using energies computed via B3LYP and GCN-ANN (Figure 6) shows that the correlation is moderate. However, the distribution of predictions and B3LYP-computed energies for LUMO, HOMO, and χ values show the presence of obvious outliers, which leads to a skewed distribution for each case. The neurotransmitter data set includes a few quaternary ammonium compounds, all of which have HOMO and LUMO energies toward or past the extremes represented in the train-test-validation set. Moreover, there are no fundamental quaternary ammonium compounds in the train-test-validation set for the GCN-ANN to learn features from. Hence, these quaternary ammonium compounds are the outliers. However, as was mentioned before, the correlation between B3LYP and GCN-ANN derived η values was quite strong. The absence of these outliers in Figure 6d is because η represents the difference between the HOMO and LUMO energies and hence the more negative estimations in B3LYP-computed energies for HOMO and LUMO cancel out. Therefore, the preservation of η demonstrates the model’s ability to implicitly learn the HOMO – LUMO gap. The neurotransmitter data set includes six quaternary ammonium compounds, all of which exhibited deviation from the model and were subsequently classified as outliers in the regression analysis through residual calculations (Figure S4) for the GCN-ANN-predicted energies of HOMO, LUMO, and χ.

**Figure 6:**
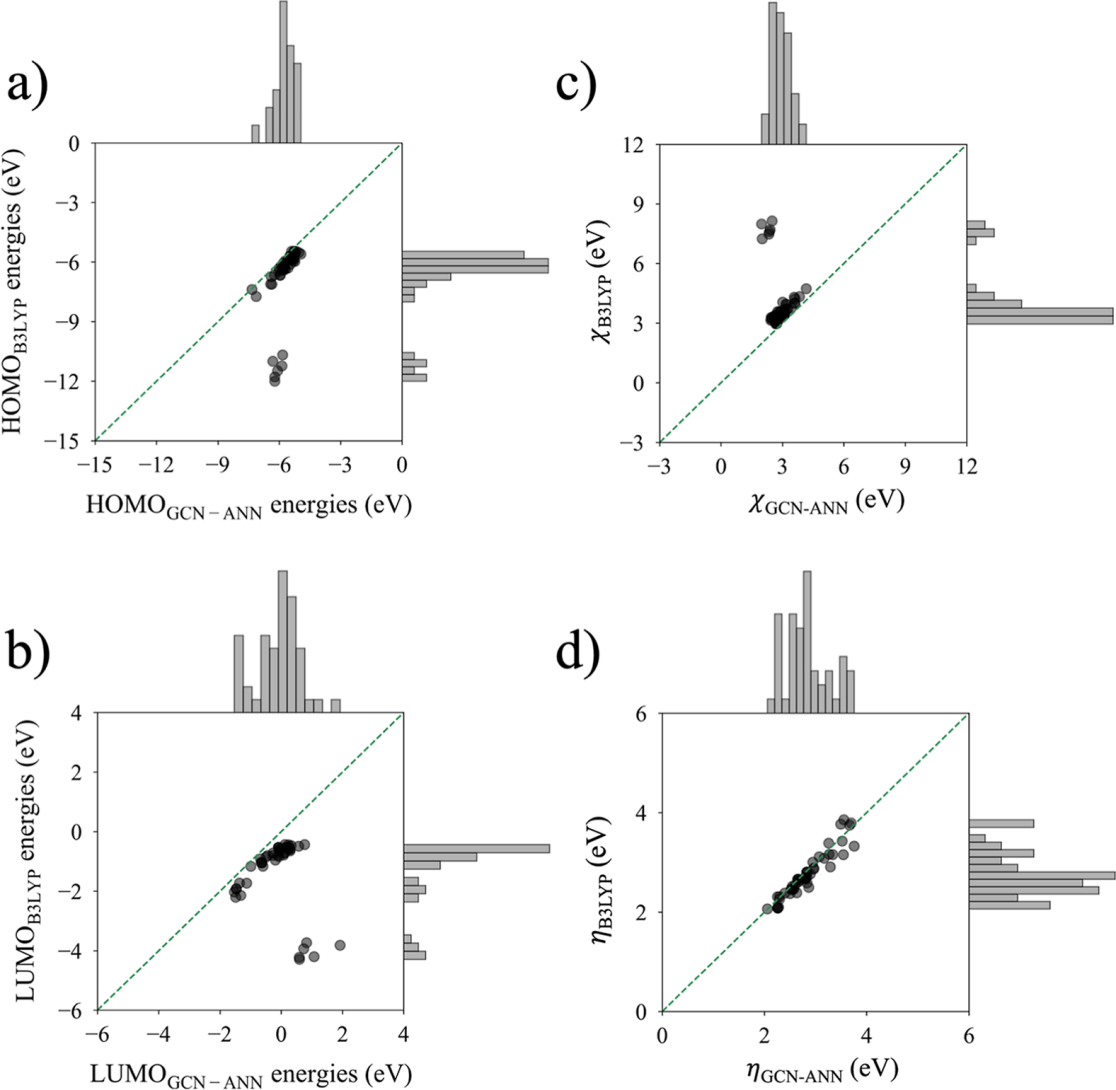
Plots GCN-ANN-predicted (a) HOMO energies, (b) LUMO energies, (c) the electronegativity (χ) (calculated using eq 1) values, and (d) the hardness values (η) (calculated using eq 2) against the B3LYP-computed values (eq 2). The calculated energies are in eV. Marginal histograms show GCN-ANN-predicted and B3LYP-computed distributions for each of the aforementioned variables.

### Model Generalizability

The presence of outliers within the GCN-ANN predictions of the neurotransmitter data set warrants a discussion on model generalizability with respect to chemical space. In Figure 6a–c, six outliers representing quaternary ammonium compounds are apparent. These six compounds with these six compounds have a positively charged ammonium group and are dissimilar from the other compounds in the neurotransmitter data set. This observation indicates that these outliers are the result of a discontinuous data set of molecules rather than a systematic limitation of the model. Specifically, the lack of quaternary ammoniums in the training set results in the inability for the model to extrapolate the extremely outlying HOMO and LUMO energies of these compounds. Thus, the training set does not adequately account for features present in the substantially dissimilar chemical space containing the quaternary ammonium compounds. For example, the range of the HOMO and LUMO energies in the entire data set,^73^ reported by the Pereira et al.,^3^ spans from −9.57 to −2.60 eV and −4.35 to 2.81 eV, respectively. On the other hand, the quaternary ammoniums compounds have average HOMO and LUMO energies centered at approximately −12 and −4 eV, respectively. Still, the preservation of the HOMO – LUMO gap (2 × η) demonstrates that the model is able to learn the foundational molecular principles across the entire chemical space despite these outliers.

### Binding Affinity Toward Neuroreceptors

The chemically hard and soft regions in the human brain was mapped earlier by Kobayashi and Terao using the chemical nature of neurotransmitters and the receptors they bind.^36^ It is known that serotonin, released by serotonergic neurons (B1–B9), and noradrenaline, released by noradrenergic neurons (A1–A7), are both unmyelinated. The receptors for serotonin and noradrenaline are found in regions of the brain that are chemically soft, including the midbrain, medulla oblongata, and spinal cord. To explore how the hardness of neurochemicals influences their response toward neuroreceptors, the binding affinity of 45 neurochemicals (28 neurotransmitters and 17 antidepressants) to four neuroreceptors—serotonin 1A or 5-HT1A receptor,^81^ noradrenaline transporter or NAT,^82^ muscarinic acetylcholine or M3R receptor,^83^ and γ-aminobutyric acid or GABA_A_ receptor^84^—were computed. The active sites for these receptors and the ligand (neurochemical) bound to respective active sites are shown in Figure 7. The binding affinity was expressed as the binding energy due to the association between a neurotransmitter (ligand) and the receptor protein—a negative quantity indicating the thermodynamic favorability of the association. The affinity for each ligand in the active site was computed for the top-ranking docking poses (Table S3). The computed binding affinity (Figure 8) shows that neurotransmitters as well as antidepressants exhibited a similar trend for 5-HT1A, NAT, and M3R receptors. The GABA_A_ receptor does not exhibit any affinity toward antidepressants as indicated by the huge positive (5–25 kcal/mol) energy values.

**Figure 7:**
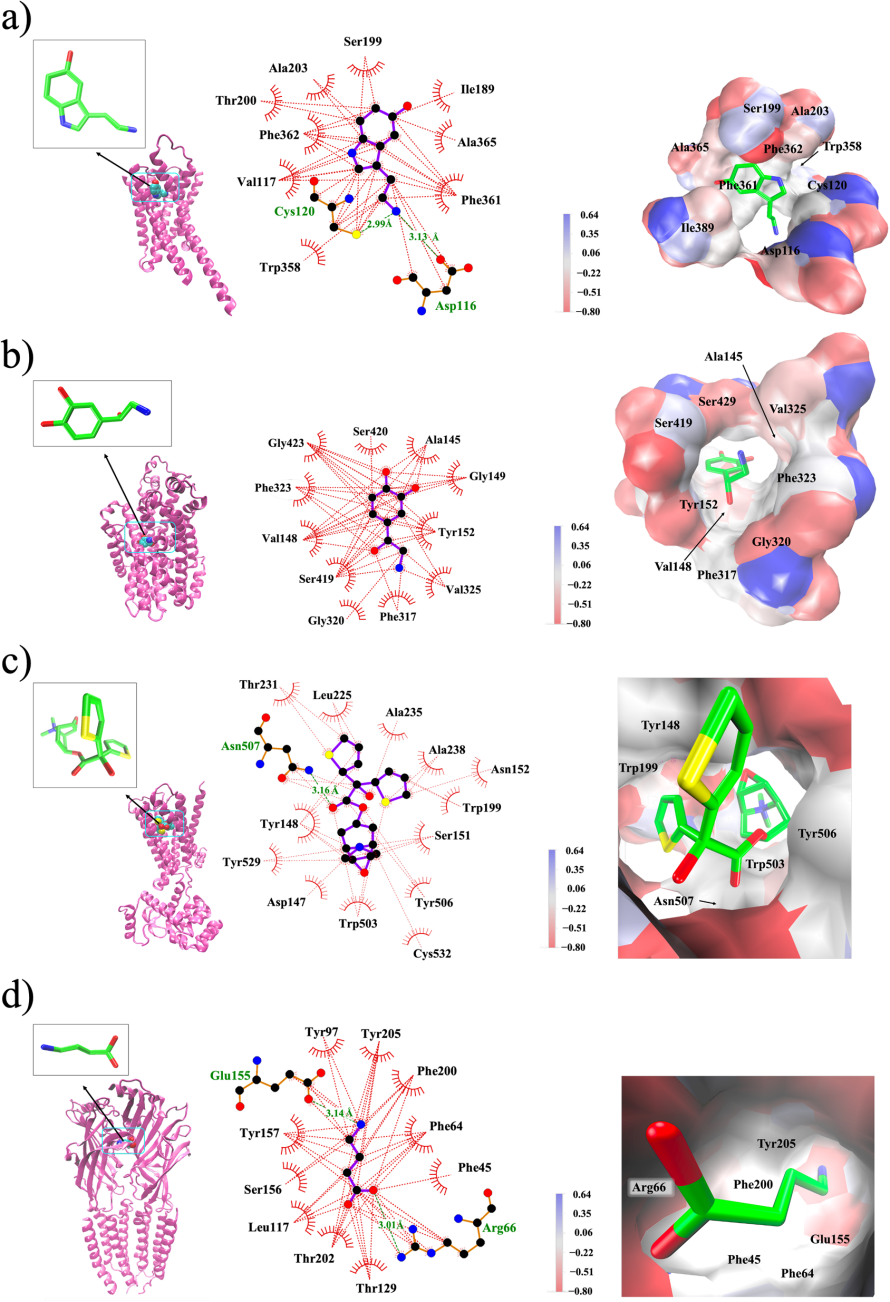
Illustration of the active site of neuroreceptors with bound ligands. From left to right: the 3D cartoon representation of the polypeptide with ligands shown in the inset, active site residues within 6 Å radius of the bound ligand, and the electrostatic potential map of the active site. (a) serotonin-bound serotonin 1A or 5-HT1A receptor (PDB code: 7e2y),^81^ (b) noradrenaline-bound noradrenaline transporter or NAT (PDB code: 8wtv), (c) tiotropium-bound muscarinic acetylcholine or M3R receptor (PDB code: 4u14), and (d) γ-aminobutyric acid-bound GABA_A_ receptor (PDB code: 8g5f). The active site-bound ligands are shown in licorice, with carbon, oxygen, and nitrogen atoms in green, red, and blue, respectively. Hydrogen atoms are omitted for clarity.

**Figure 8:**
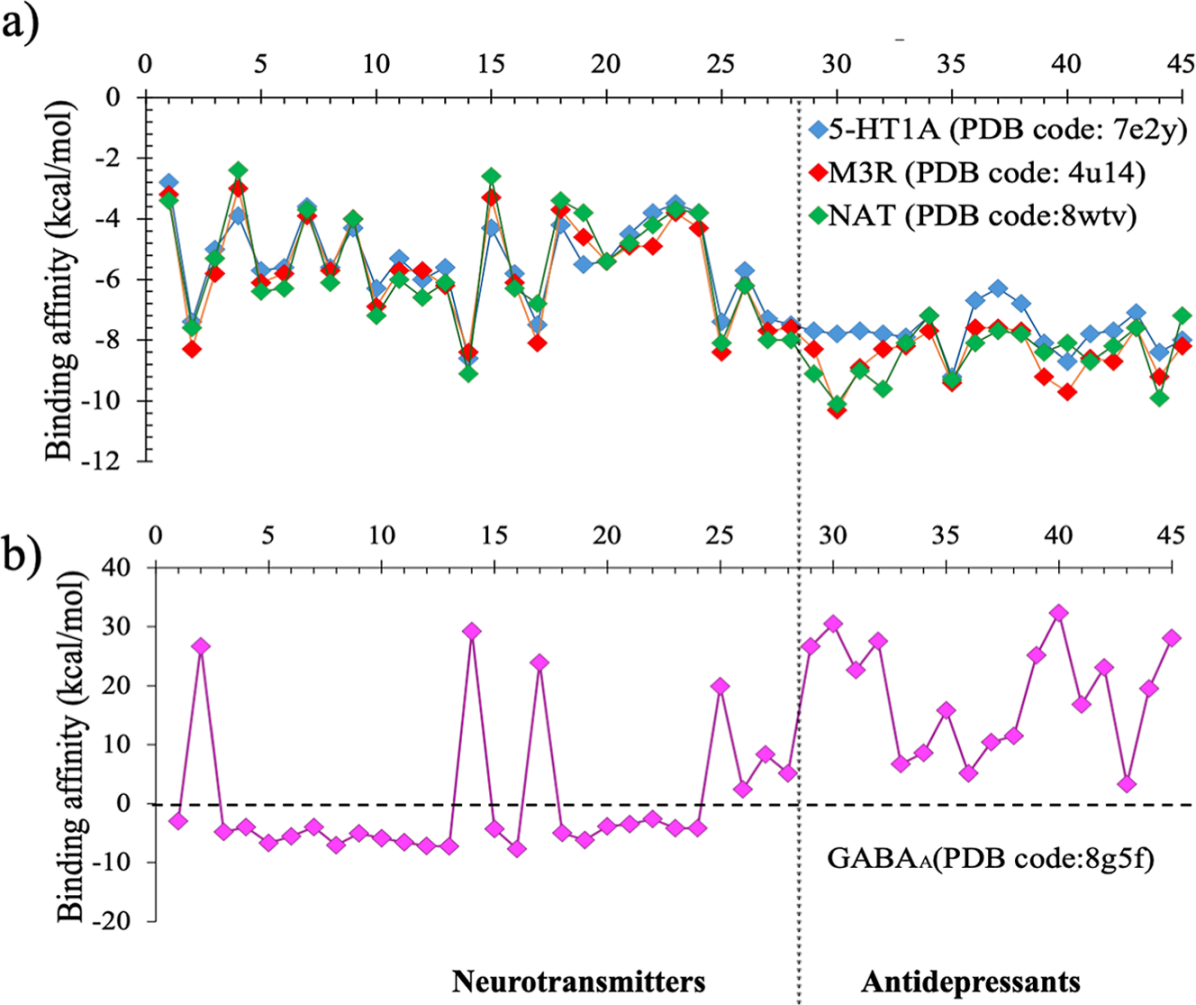
Binding affinity trend observed for neurotransmitters and antidepressants toward neuroreceptors (a) 5-HT1A receptor (PDB code: 7e2y), M3R receptor (PDB code: 4u14), NAT (PDB code: 8wtv); (b) GABA_A_ receptor (PDB code: 8g5f).

### Hardness and Binding Affinity

Since the binding trends of neurochemicals studied for the three neuroreceptors 5-HT1A,^81^ NAT,^82^ and M3R^83^ (Figure 8a) exhibit a considerable similarity, if the hardness impacted their binding, then the variation of hardness with binding affinity would also produce similar trends. Therefore, the GCN-ANN-derived hardness (calculated using eq 2) of these neurochemicals were plotted against their affinities to a specific receptor molecule (Figure 9). As evident in Figure 9a–c, the variation of binding affinity and hardness for the three receptors—5-HT1A receptor (PDB code: 7e2y), M3R receptor (PDB code: 4u14), and NAT (PDB code: 8wtv)—maintained similar pattern consistent to their binding trend observed in Figure 8a. This indicated that the hardness would have contributed to the binding, which in turn demonstrates that the GCN-ANN-generated HOMO and LUMO has predictability in terms of physical molecular–level interactions. As observed in Figure 9a–c, the overall linear trend, with correlation coefficients ranging from 0.7 to 0.8, indicates that binding affinity for neurochemicals increases as their hardness decreases. This indicates that these neuroreceptors possess soft active sites, which is supported by the observation that hydrophobic and aromatic residues predominantly occupy these regions (Figure 7). For the GABA_A_ receptor, several neurochemicals exhibited unfavorable binding (Figure S5) and a scrutiny of the structures of these molecules reveals that these molecules contain bulkier groups with fused ring system resulting in severe steric repulsion within the active site and hence positive values of the binding affinity. However, a linear trend is obtained from the actives (i.e., molecules with negative binding affinities) suggesting that the active site preferred chemically softer molecules (Figure S5).

**Figure 9:**
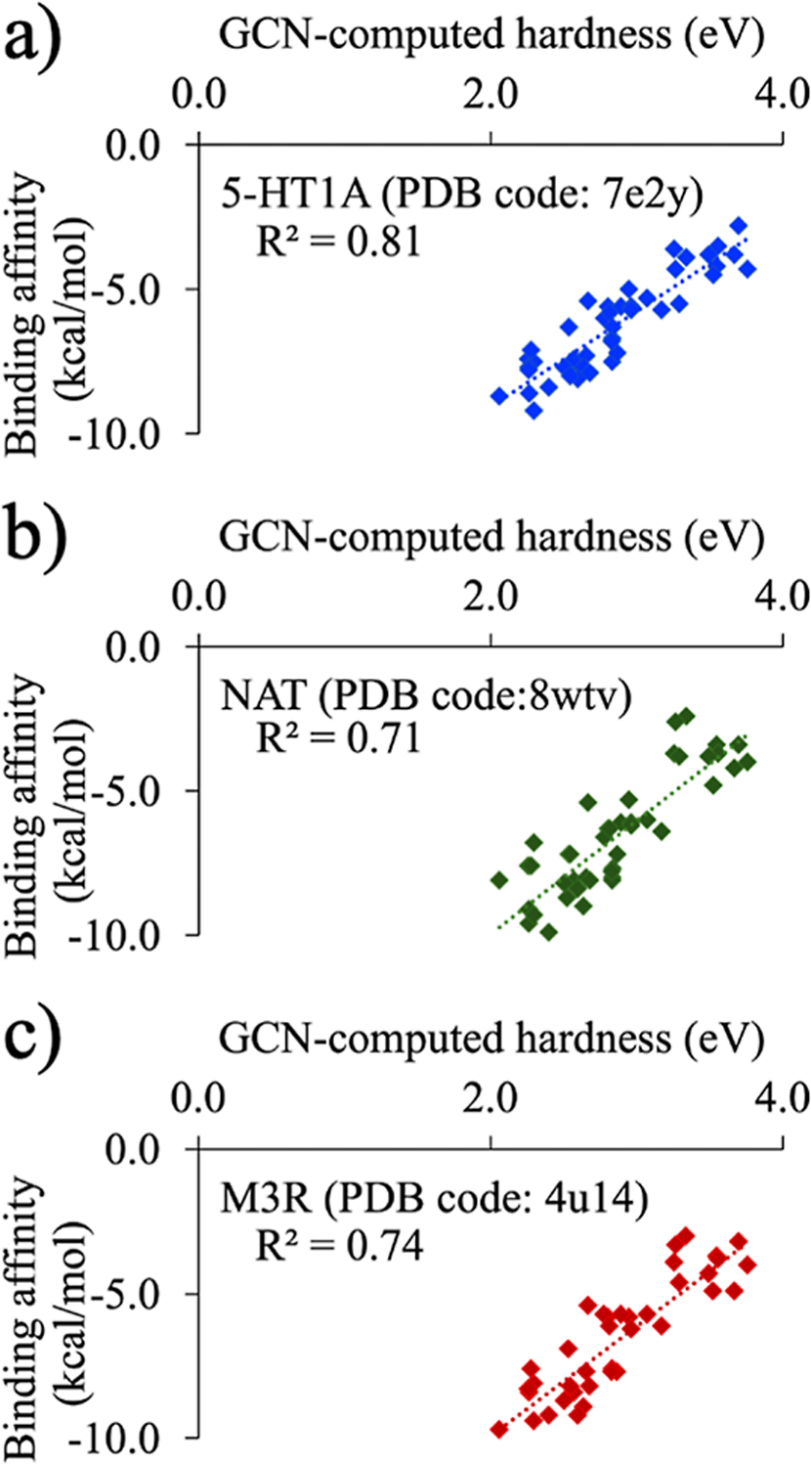
Binding affinity of 28 neurotransmitters and 17 antidepressants plotted against their GCN-ANN-computed hardness for (a) 5-HT1A receptor (PDB code: 7e2y), (b) NAT (PDB code: 8wtv), and (c) M3R receptor (PDB code: 4u14).

### Similarities in Structures Within Clusters

The plot of GCN-ANN-computed hardness versus binding affinity for M3R receptor (PDB code: 4u14) was further analyzed, especially ascertaining the clusters of molecules and their structural similarities (Figure 10). These structurally similar clusters were also identified for 5-HT1A (PDB code: 7e2y) and (b) NAT (PDB code: 8wtv) receptors (Figure S6), indicating reproducible correlation between the structural motifs of ligands and their binding affinities. As observed in the structural analysis of three distinct clusters in Figure 10, each cluster represents structurally related molecules that exhibited similarities in hardness as well as a similar binding affinity toward the active site of a neuroreceptor. This observation demonstrates that the structural basis of binding, as identified through the molecular docking, is fundamentally associated with molecular orbital of these neurochemicals (vide infra). A closer examination of the active site, located within the trans-helices bundle of the M3R receptor (Figure 11), further confirms that the hydrophobic side chains of the active sites are responsible for intermediate to high binding affinity. A subset of residues within 5 Å of the bound ligands were analyzed in detail, which exhibited that there are four Tyr (Y148, Y506, Y529, Y533), two Trp (W199, W503), and one Phe (F239) residues, indicating a soft active site center (Figure 11). This is supported by the increased binding affinity of molecules of lower hardness by the active site as observed in Figure 9.

**Figure 10:**
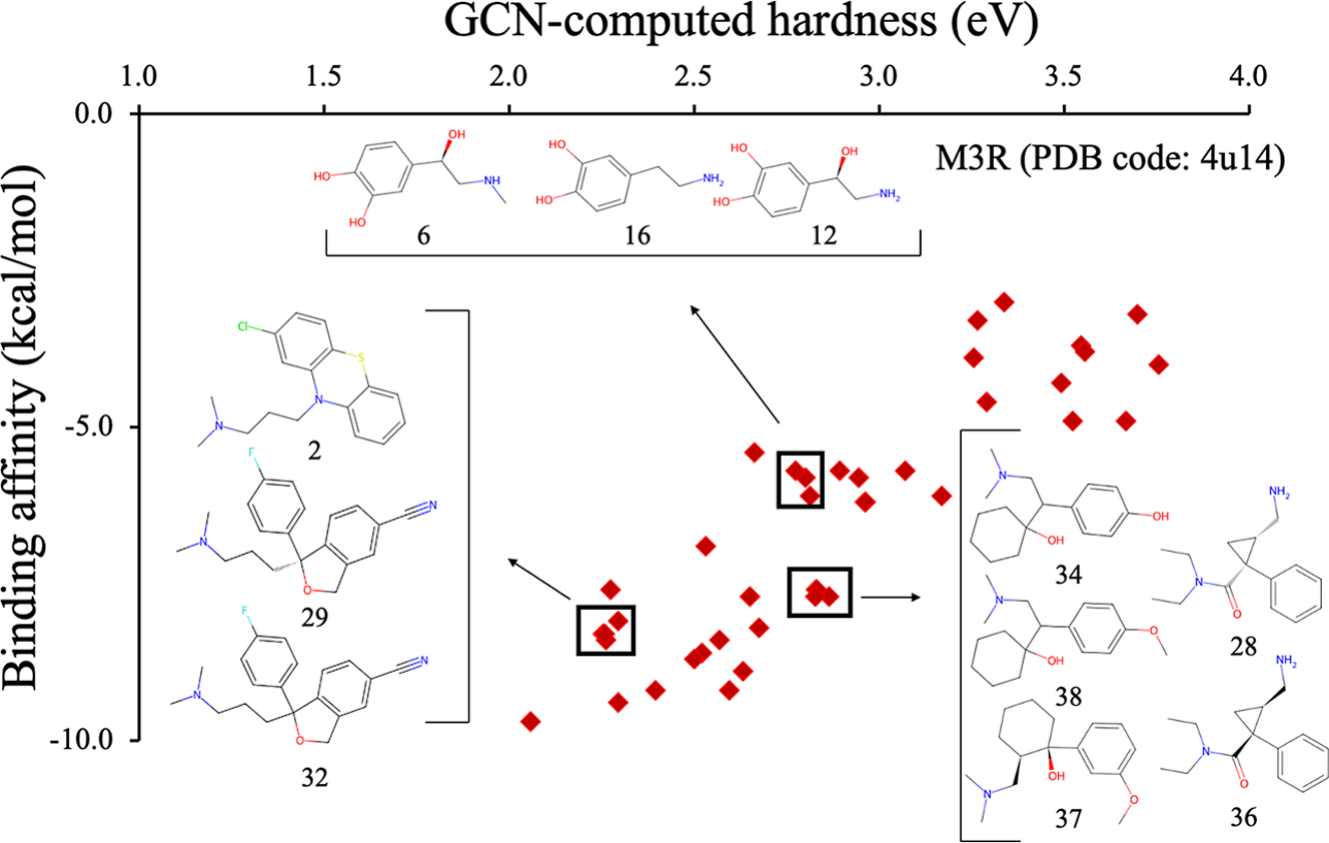
Chemical structure of molecules in three clusters observed in the GCN-ANN-calculated hardness (η in eV) vs binding affinity (in kcal/mol) plot of neurochemicals docked into the M3R receptor (PDB code: 4u14). Substructural similarity is evident in the 2D structures of overlapping molecules grouped within rectangles.

**Figure 11:**
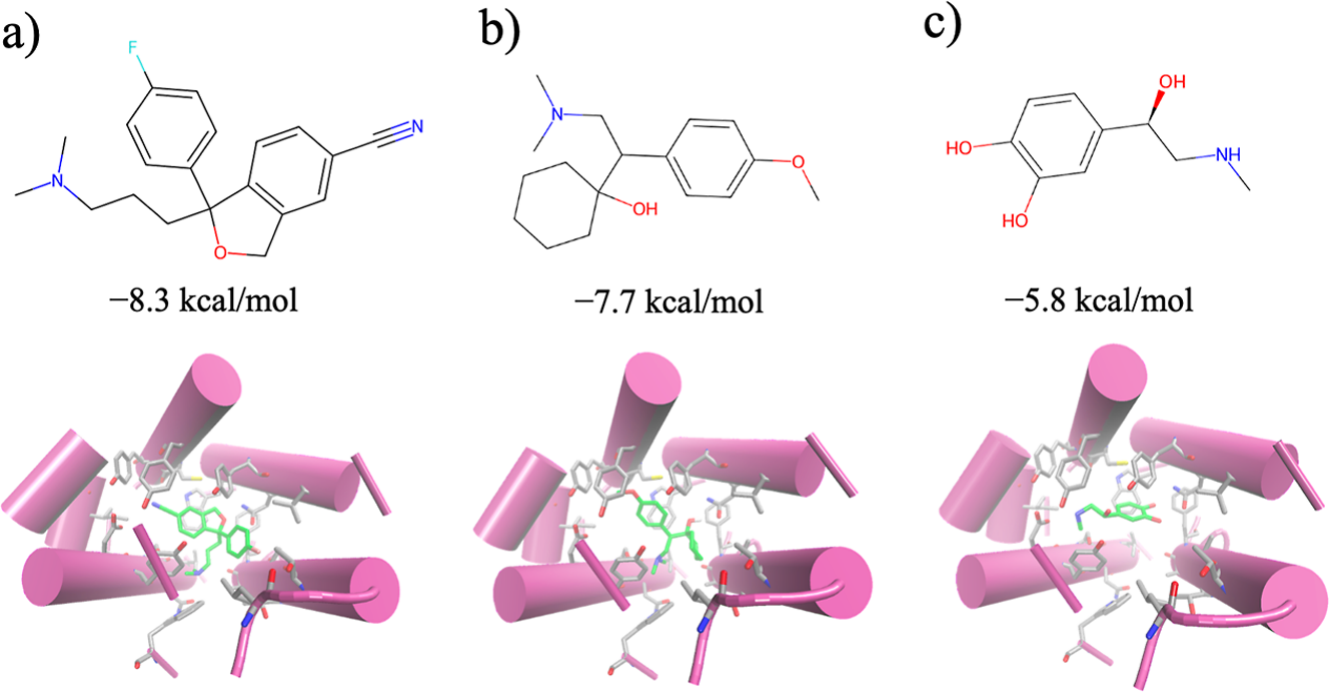
Binding poses of three representative ligands taken from the three clusters shown in Figure 10 and docked into the active site of M3R receptor (PDB code: 4u14): (a) escitalopram, (b) venlafaxine, (c) adrenaline. The binding affinities (kcal/mol) are displayed for each bound.

### Maximal Activation-Based Substructure in the Fingerprint and Kohn–Sham HOMO Electron Density

An interesting finding from the present study is the correlation between the molecular orbital space and the molecular substructures identified by the GCN-ANN algorithm. During the generation of fingerprints for molecules, certain substructures had a greater impact on the model’s predictability of the HOMO and LUMO energies. Thus, if the ML-based GCN-ANN model accurately explains the physical origin of the molecular orbital space, then atoms and bonds in a substructure identified from the neural fingerprint would correspond to the Kohn–Sham electron density of the frontier molecular orbitals. To explore the hypothesis, the substructures determined from the maximal fingerprint activation were compared with the Kohn–Sham HOMO electron density for ligands, which exhibited stronger binding for the neuroreceptors 5-HT1A, M3R, and NAT. The binding pocket of 5-HT1A with two strong binding antidepressants—atomoxetine and duloxetine—was examined (Figure 12) and related with their frontier molecular orbitals.

**Figure 12:**
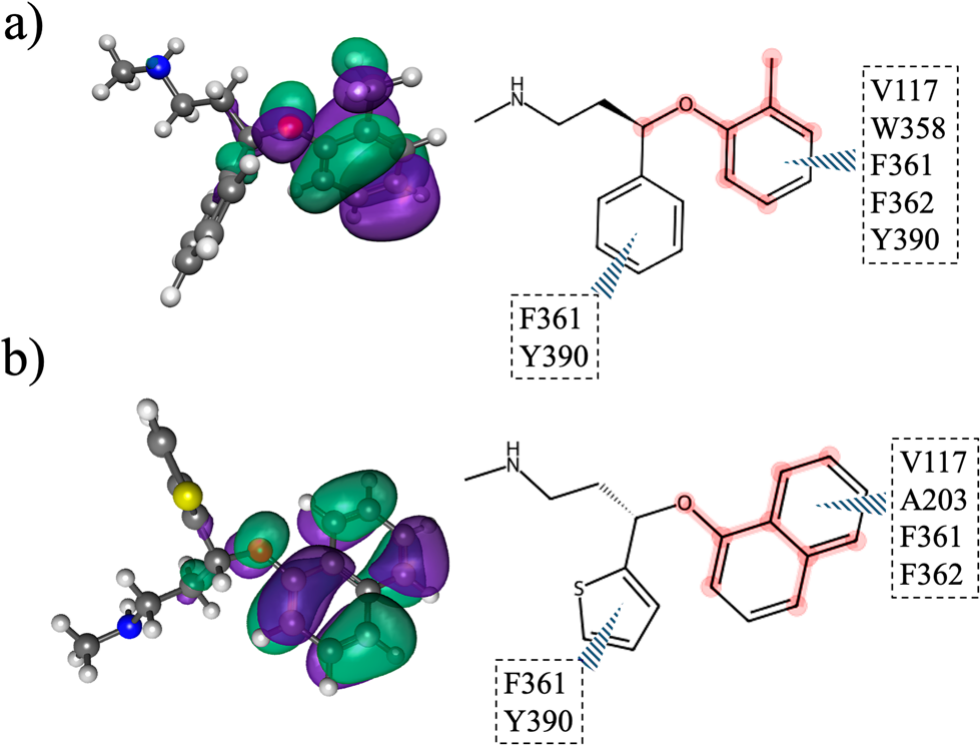
Localization of the HOMO and the corresponding substructure, as determined by maximal activation in the GCN-ANN model, for two neurotransmitters in their bound conformation at the 5-HT1A receptor active site (PDB code: 7e2y): (a) atomoxetine and (b) duloxetine. Both molecules exhibited strong binding toward 5-HT1A, with a binding affinity of −7.9 and −9.4 kcal/mol, respectively. The left panel depicts the localization of the electron density as colored surfaces on a molecule with the green and purple colors signifying the opposite signs of the wave function. The right panel shows red-highlighted segments on the 2D molecular structure representing regions with maximum fingerprint activation, while the interactions between a molecular fragment and active site residues (indicated with one-letter amino acid code) are illustrated using dashed lines.

For both antidepressants, the localization of the Kohn–Sham HOMO electron density on each antidepressant coincided with their respective GCN-ANN-derived substructure (highlighted in red, Figure 12). The importance of the localized HOMO electron density can be rationalized from its interactions within the active site dominated by hydrophobic side chains (Figure 12). In particular, the substructures of both atomoxetine and duloxetine, where HOMOs are localized, were found to interact with several active site hydrophobic residues. This was also supported by a computational experiment, which was carried out by removing the tolyl group in atomoxetine and the naphthyl group in duloxetine and then performing the docking study. There was a 3–5 kcal/mol decrease in the binding affinities for the altered molecules; the observed binding affinities were −5.5 kcal/mol for atomoxetine and −5.3 kcal/mol for duloxetine. This demonstrated that the substructures identified in the neural fingerprint generation attributed to HOMO density, which in turn is consequential for antidepressant binding.

## Conclusion

Millions worldwide are affected by depression and the effectiveness of antidepressants is often linked to their interactions with neuroreceptors. Laboratory testing and clinical studies of these interactions are expensive and time-consuming, which makes the computational approach an increasingly important step for drug discovery. Using a graph convolutional network, a neural fingerprint-based architecture (GCN-ANN) has been developed for HOMO–LUMO prediction using B3LYP-computed HOMO–LUMO data of >110,000 molecules. The evaluation of the GCN-ANN model was carried out using standard statistical parameters, all of which demonstrated significant accuracy against the data. This model was used to gain a molecular orbital-based understanding of the binding affinity of various neurochemicals for different neurotransmitters. The binding affinity to four different receptors was carried out, with three of them exhibiting a trend of tighter binding as hardness (η) decreased. The fourth receptor exhibited a similar pattern among the actives; moreover, the docking study revealed that inactives (i.e., showing no binding affinity) included several neurotransmitters and all antidepressants with bulkier ring systems. The plot of binding affinity versus chemical hardness indicates that structurally similar molecules produced similar binding affinity. Furthermore, the Kohn–Sham-HOMO density in two active molecules (tight binding or higher binding affinity) was found to be superimposable to the maximal activation-based substructure identified in the GCN-ANN model. Finally, the significance of the substructure was revealed from a computational docking experiment, where the removal of fragments corresponding to the substructures lead to significant reduction in the binding affinity to the neuroreceptor. This confirms that the GCN-ANN model is capable of capturing the core chemical physics that can be readily translated to rational antidepressant design.

## Supplementary Material



## Data Availability

All data including the codes developed for the presented model, the machine-readable input for molecules, and frontier MO energies needed to reproduce the essential results are available at https://github.com/rivmons/gcn-frontiermo.

## Notes

Contains data tables of electronegativity and hardness values for 45 neurochemicals along with their binding affinities toward four neuroreceptors. Additionally, figures with electronegativity and hardness values computed under two different theoretical assumptions are included (PDF)
