# Supplementary material for: Graph Convolutional Neural Network-Enabled Frontier Molecular Orbital Prediction: A Case Study with Neurotransmitters and Antidepressants
Source: J Chem Inf Model. 2025 Jul 17;65(14):7447–62. doi: 10.1021/acs.jcim.5c00724 (PMC12308792; doi:10.1021/acs.jcim.5c00724)

07/08/2025 **SUPPORTING INFORMATION**

Graph Convolutional Neural Network-Enabled Frontier Molecular Orbital Prediction: A Case Study with Neurotransmitters and Antidepressants

Rivaaj Monsia,† Stewart C. Gundry,† Molly L. Mohr, Macey A. Smith, Sudeep Bhattacharyya*, and Sanchita Hati*

Department of Chemistry and Biochemistry, University of Wisconsin – Eau Claire, Eau Claire, Wisconsin, 54702, United States

*To whom correspondence should be addressed: S.B.: phone 715-836-2278, email: bhattas@uwec.edu; S.H.: phone: 715-836-3850; email: hatis@uwec.edu; fax: 715-836-4979.

†Equally contributed to this study

Table S1. Absolute electronegativity and absolute hardness of neurotransmitters in eV, computed at the level of HF (6-31G(d,p); eqs. 1, 2) and DFT (eqs. 5, 6) using B3LYP, wb97xd, and M06-2x functionals and 6-311++G(d,p) basis set.

	**Neurotransmitters**	**B3LYP**		**ωb97xd**		**M06-2x**		**HF**	
		χ	η	χ	η	χ	η	χ	η
**1**	Choline	8.70	5.72	8.48	5.98	8.68	5.96	7.77	8.39
**2**	Chlorpromazine	3.26	3.44	3.09	3.78	3.46	3.54	2.10	5.31
**3**	Barbital	4.88	4.66	4.93	4.95	5.07	5.03	4.23	7.55
**4**	Aspartic acid	4.83	4.62	4.71	4.77	4.81	4.90	3.76	7.84
**5**	Amphetamine	3.71	4.17	3.63	4.50	3.77	4.48	2.37	6.34
**6**	Adrenaline	3.61	3.95	3.34	4.13	3.48	4.05	2.03	6.00
**7**	Acetylcholine	8.09	5.07	7.87	5.33	8.06	5.30	7.33	8.12
**8**	Tyramine	3.58	3.94	3.56	4.41	3.69	4.45	2.14	6.03
**9**	Taurine	4.78	3.32	4.70	2.03	4.89	3.61	3.76	6.04
**10**	Serotonin	3.12	3.46	2.91	3.72	3.06	3.74	1.90	5.50
**11**	Phenethylamine	3.75	4.15	3.91	4.74	3.79	4.57	2.33	6.38
**12**	Noradrenaline	3.60	3.85	3.44	4.06	3.19	4.50	2.08	6.05
**13**	m-Tyramine	3.82	4.17	3.64	4.47	3.63	4.66	2.19	6.14
**14**	Haloperidol	3.83	3.26	3.37	3.74	4.03	3.24	3.13	5.55
**15**	Glutamic acid	4.05	2.86	3.91	3.00	3.74	2.71	3.03	5.62
**16**	Dopamine	3.50	3.84	3.42	4.16	3.53	4.19	1.97	6.03
**17**	Diazepam	4.40	3.58	4.39	3.77	4.51	3.78	3.36	5.54
**18**	γ-aminobutyric acid	4.04	2.49	3.92	2.62	4.11	2.74	2.82	5.05
**19**	Taurocyamine	4.07	4.14	3.90	4.31	4.55	3.86	2.26	7.54
**20**	Nicotine	3.51	3.90	3.27	4.19	3.50	4.14	2.78	6.26
**21**	Muscarine	7.27	4.68	7.10	4.79	7.30	4.75	6.68	7.77
**22**	$CH_3COOCH_2CH_2N^+(C_2H_5)_3$	7.82	5.12	7.61	5.39	7.77	5.40	6.90	8.48
**23**	$CH_3COOCH_2N^+(CH_3)(C_2H_5)_2$	8.81	4.96	8.07	5.79	8.26	5.78	7.39	8.56
**24**	$CH_3COOCH_2CH_2N^+(CH_3)_2(C_2H_5)$	7.98	5.10	7.75	5.35	7.95	5.35	7.16	8.03
**25**	Impramine	3.17	3.52	3.05	3.88	3.05	4.04	2.04	5.85
**26**	Oxotremorine	3.54	3.85	3.15	3.92	3.37	3.92	2.14	7.52
**27**	Fluoxetine	4.62	3.51	2.53	5.50	3.75	4.43	2.73	6.14
**28**	Milnacipran	3.70	3.70	3.70	4.31	3.86	4.33	2.60	6.25

Table S2. Absolute electronegativity and absolute hardness of antidepressants in eV, computed at the level of HF (6-31G(d,p); eqs. 1, 2) and DFT (eqs. 5, 6) using B3LYP, ωb97xd, and M06-2x functionals (basis set: 6-311++G(d,p)).

	**Antidepressants**	**B3LYP**		**ωb97xd**		**M06-2x**		**HF**	
		χ	η	χ	η	χ	η	χ	η
**29**	Citalopram (Celexa)	3.97	3.43	3.97	3.59	4.09	3.63	3.37	5.66
**30**	Sertraline (Zoloft)	3.95	3.60	3.91	3.80	3.97	3.92	2.81	5.94
**31**	Paroxetine (Paxil)	3.33	3.70	3.22	3.96	3.39	4.00	2.15	5.63
**32**	Escitalopram (Lexapro)	4.85	4.45	4.50	4.35	3.97	3.74	3.38	5.65
**33**	Atomoxetine (Strattera)	3.47	3.82	3.41	4.17	3.58	4.19	2.14	5.96
**34**	Desvenlafaxine (Pristiq)	3.32	3.62	3.09	3.83	3.26	3.80	1.96	6.00
**35**	Duloxetine (Cymbalta)	3.49	3.54	3.52	3.71	3.66	3.77	2.20	5.25
**36**	Levomilnacipran (Fetzima)	3.73	4.03	3.67	4.40	3.83	4.40	2.42	6.26
**37**	Tramadol (Ultram)	3.61	3.96	3.11	3.97	3.20	3.81	2.22	6.11
**38**	Venlafaxine (Effexor)	3.34	3.64	3.07	3.79	3.24	3.80	2.02	5.97
**39**	Amitriptyline (Elavil)	3.42	3.56	3.42	3.82	3.58	3.80	2.44	5.84
**40**	Amoxapine (Asendin)	3.72	3.09	3.70	3.23	3.86	3.28	2.14	5.95
**41**	Desipramine (Norpramin)	3.22	3.57	3.09	3.84	3.25	3.84	2.05	5.84
**42**	Doxepin (Silenor)	3.48	3.46	3.60	3.83	3.77	3.84	2.42	5.58
**43**	Bupropion (Wellbutrin)	4.28	3.39	4.23	3.47	4.35	3.27	3.58	5.76
**44**	Vortioxetine (Trintellix)	3.18	3.52	3.10	3.59	3.18	3.65	2.20	5.66
**45**	Mirtazapine (Remeron)	3.21	3.79	3.16	3.92	3.30	3.95	2.11	5.70

Table S3. Absolute electronegativity (χ, eq. 1) and absolute hardness (η, eq. 2) of neurotransmitters in eV, computed at the level of DFT using B3LYP (basis set: 6-311++G(d,p) basis set) and GC-ANN.

	**Neurotransmitters**	**B3LYP**		**GCN-ANN**	
		χ	η	χ	η
**1**	Choline	7.99	3.79	1.98	3.70
**2**	Chlorpromazine	3.22	2.31	2.80	2.25
**3**	Barbital	4.73	3.00	4.16	2.95
**4**	Aspartic acid	3.95	3.16	3.18	3.34
**5**	Amphetamine	3.59	3.08	2.85	3.17
**6**	Adrenaline	3.19	2.67	2.46	2.80
**7**	Acetylcholine	7.61	3.39	2.36	3.26
**8**	Tyramine	3.35	2.76	2.58	2.89
**9**	Taurine	4.06	3.33	3.01	3.76
**10**	Serotonin	3.15	2.45	2.43	2.53
**11**	Phenethylamine	3.55	3.11	2.88	3.07
**12**	Noradrenaline	3.26	2.68	2.46	2.77
**13**	m-Tyramine	3.43	2.87	2.68	2.96
**14**	Haloperidol	4.22	2.08	3.61	2.26
**15**	Glutamic acid	3.95	3.16	3.17	3.26
**16**	Dopamine	3.29	2.68	2.44	2.81
**17**	Diazepam	4.34	2.31	3.83	2.29
**18**	γ-aminobutyric acid	3.59	3.16	2.77	3.55
**19**	Taurocyamine	3.55	2.91	2.89	3.29
**20**	Nicotine	3.58	2.62	3.09	2.66
**21**	Muscarine	7.24	3.43	2.01	3.52
**22**	$CH_3COOCH_2CH_2N^+(C_2H_5)_3$	7.48	3.75	2.33	3.67
**23**	$CH_3COOCH_2N^+(CH_3)(C_2H_5)_2$	8.14	3.86	2.49	3.56
**24**	$CH_3COOCH_2CH_2N^+(CH_3)_2(C_2H_5)$	7.70	3.77	2.39	3.49
**25**	Impramine	3.00	2.47	2.69	2.57
**26**	Oxotremorine	3.38	2.89	2.72	2.96
**27**	Fluoxetine	3.70	2.66	3.24	2.65
**28**	Milnacipran	3.43	2.81	3.08	2.83

Table S4. Absolute electronegativity (χ, eq. 1) and absolute hardness (η, eq. 2) of antidepressants in eV, computed at the level of DFT using B3LYP (basis set: 6-311++G(d,p) basis set) and GC-ANN.

	**Antidepressants**	**B3LYP**		**GCN-ANN**	
		χ	η	χ	η
**29**	Citalopram (Celexa)	4.00	2.09	3.60	2.26
**30**	Sertraline (Zoloft)	3.70	2.67	3.10	2.65
**31**	Paroxetine (Paxil)	3.23	2.39	2.68	2.63
**32**	Escitalopram (Lexapro)	4.00	2.09	3.60	2.26
**33**	Atomoxetine (Strattera)	3.34	2.62	3.01	2.68
**34**	Desvenlafaxine (Pristiq)	2.96	2.50	2.67	2.86
**35**	Duloxetine (Cymbalta)	3.43	2.26	3.11	2.29
**36**	Levomilnacipran (Fetzima)	3.43	2.81	3.08	2.83
**37**	Tramadol (Ultram)	3.12	2.59	2.71	2.83
**38**	Venlafaxine (Effexor)	3.15	2.71	2.62	2.83
**39**	Amitriptyline (Elavil)	3.39	2.54	3.03	2.59
**40**	Amoxapine (Asendin)	3.79	2.07	3.44	2.06
**41**	Desipramine (Norpramin)	2.99	2.47	2.73	2.52
**42**	Doxepin (Silenor)	3.53	2.37	3.11	2.50
**43**	Bupropion (Wellbutrin)	4.30	2.10	3.57	2.27
**44**	Vortioxetine (Trintellix)	3.18	2.38	2.82	2.40
**45**	Mirtazapine (Remeron)	3.32	2.52	2.88	2.54

Table S5. The binding affinity (kcal/mol) for the neurotransmitters for the receptors 5-HT1A (PDB code: 7e2y), M3R (PDB code: 4u14), $GABA_A$ (PDB code: 8g5f), and NAT (PDB code: 8wtv).

	Neurotransmitters	5-HT1A	NAT	M3R	$GABA_A$
1	Choline	–2.8	–3.4	–3.2	–3.0
2	Chlorpromazine	–7.4	–7.6	–8.3	26.7
3	Barbital	–5.0	–5.3	–5.8	–4.8
4	Aspartic acid	–3.9	–2.4	–3.0	–4.0
5	Amphetamine	–5.7	–6.4	–6.1	–6.7
6	Adrenaline	–5.6	–6.3	–5.8	–5.6
7	Acetylcholine	–3.6	–3.7	–3.9	–4.0
8	Tyramine	–5.6	–6.1	–5.7	–7.1
9	Taurine	–4.3	–4.0	–4.0	–5.1
10	Serotonin	–6.3	–7.2	–6.9	–5.9
11	Phenethylamine	–5.3	–6.0	–5.7	–6.6
12	Noradrenaline	–6.0	–6.6	–5.7	–7.2
13	*m*–Tyramine	–5.6	–6.1	–6.2	–7.3
14	Haloperidol	–8.6	–9.1	–8.4	29.2
15	Glutamic acid	–4.3	–2.6	–3.3	–4.3
16	Dopamine	–5.8	–6.3	–6.1	–7.7
17	Diazepam	–7.5	–6.8	–8.1	23.9
18	γ–Aminobutyric acid	–4.2	–3.4	–3.7	–5.0
19	Taurocyamine	–5.5	–3.8	–4.6	–6.2
20	Nicotine	–5.4	–5.4	–5.4	–3.9
21	Muscarine	–4.5	–4.8	–4.9	–3.5
22	$CH_3COOCH_2CH_2N^+(C_2H_5)_3$	–3.8	–4.2	–4.9	–2.6
23	$CH_3COOCH_2N^+(CH_3)(C_2H_5)_2$	–3.5	–3.7	–3.8	–4.2
24	$CH_3COOCH_2CH_2N^+(CH_3)_2(C_2H_5)$	–3.8	–3.8	–4.3	–4.2
25	Impramine	–7.4	–8.1	–8.4	19.9
26	Oxotremorine	–5.7	–6.2	–6.2	2.4
27	Fluoxetine	–7.3	–8.0	–7.7	8.3
28	Milnacipran	–7.5	–8.0	–7.6	5.1

Table S6. The binding affinity (kcal/mol) for the antidepressants for the receptors 5-HT1A (PDB code: 7e2y), M3R (PDB code: 4u14), NAT (PDB code: 8wtv), and $GABA_A$ (PDB code: 8g5f).

	**Antidepressants**	**5-HT1A**	**NAT**	**M3R**	**$GABA_A$**
**29**	Citalopram (Celexa)	–7.7	–9.1	–8.3	26.7
**30**	Sertraline (Zoloft)	–7.8	–10.1	–10.3	30.5
**31**	Paroxetine (Paxil)	–7.7	–9.0	–8.9	22.7
**32**	Escitalopram (Lexapro)	–7.8	–9.6	–8.3	27.6
**33**	Atomoxetine (Strattera)	–7.9	–8.1	–8.2	6.7
**34**	Desvenlafaxine (Pristiq)	–7.2	–7.2	–7.7	8.6
**35**	Duloxetine (Cymbalta)	–9.2	–9.3	–9.4	15.8
**36**	Levomilnacipran (Fetzima)	–6.7	–8.1	–7.6	5.1
**37**	Tramadol (Ultram)	–6.3	–7.7	–7.6	10.4
**38**	Venlafaxine (Effexor)	–6.8	–7.8	–7.7	11.5
**39**	Amitriptyline (Elavil)	–8.1	–8.4	–9.2	25.2
**40**	Amoxapine (Asendin)	–8.7	–8.1	–9.7	32.3
**41**	Desipramine (Norpramin)	–7.8	–8.7	–8.6	16.8
**42**	Doxepin (Silenor)	–7.7	–8.2	–8.7	23.1
**43**	Bupropion (Wellbutrin)	–7.1	–7.6	–7.6	3.3
**44**	Vortioxetine (Trintellix)	–8.4	–9.9	–9.2	19.5
**45**	Mirtazapine (Remeron)	–8.0	–7.2	–8.2	28.1

Table S7. A comparison of the assessment data for the GCN-ANN study (this work) and the same using Random Forest algorithm, reported by Pereira *et al.* for the three best performing descriptors (Modified distance (Md), SubstructureCount (SubC), and PubChem fingerprints). The energy values in RMSE and MAE are provided in eV.

**Energies/ ML methods**	**GCN-ANN**	**RF-Md** [a]	**RF-SubC** [a]	**RF-PubChem** [a]
	RMSE (MAE) [R^2]	RMSE (MAE) [R^2]	RMSE (MAE) [R^2]	RMSE (MAE) [R^2]
**HOMO**	0.118 (0.087) [0.966]	0.230 (0.161) [.8765]	0.240 (0.169) [.8592]	0.220 (0.155) [.8765]
**LUMO**	0.091 (0.065) [0.989]	0.281 (0.185) [.9045]	0.264 (0.179) [.9126]	0.261 (0.175) [.9151]
**Gap**	0.074 (0.056) [0.981]	0.330 (0.231) [.8852]	0.323 (0.223) [.8861]	0.303 (0.208) [.9002]

[a] Data abstracted from reference number 3, Pereira *et al.* *J. Chem. Inf. Model.* 2017*, 57* (1), 11–21.

Figure S1. Correlation between B3LYP-computed electronegativities (χ) and hardnesses (η) using two theoretical treatments described in the theory and methods sections. a) χ (eV) (using eqs. 1 and 5) and b) η (eV) (using eqs. 2 and 6). All calculations of the 45 neurochemical molecules were carried out in the gas phase using the 6-311++G(d,p) basis set.

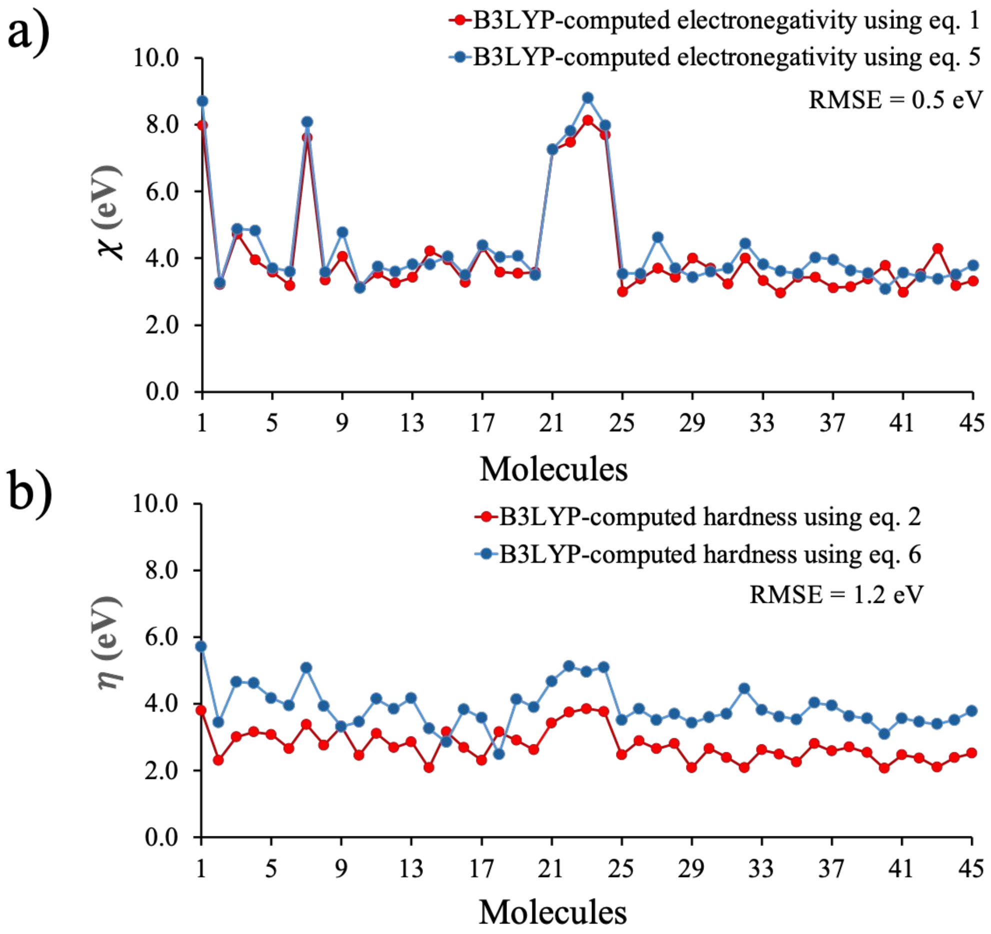

Figure S2. Correlation between B3LYP-computed electronegativities using eq. 1 and electronegativities computed using a) B3LYP (6-311++G(d,p) and eq. 5), b) ωB97XD (6-311++G(d,p) and eq. 5), c) M06-2X (6-311++G(d,p), and eq. 5), and d) HF (6-31G(d,p)) and eq. 1).

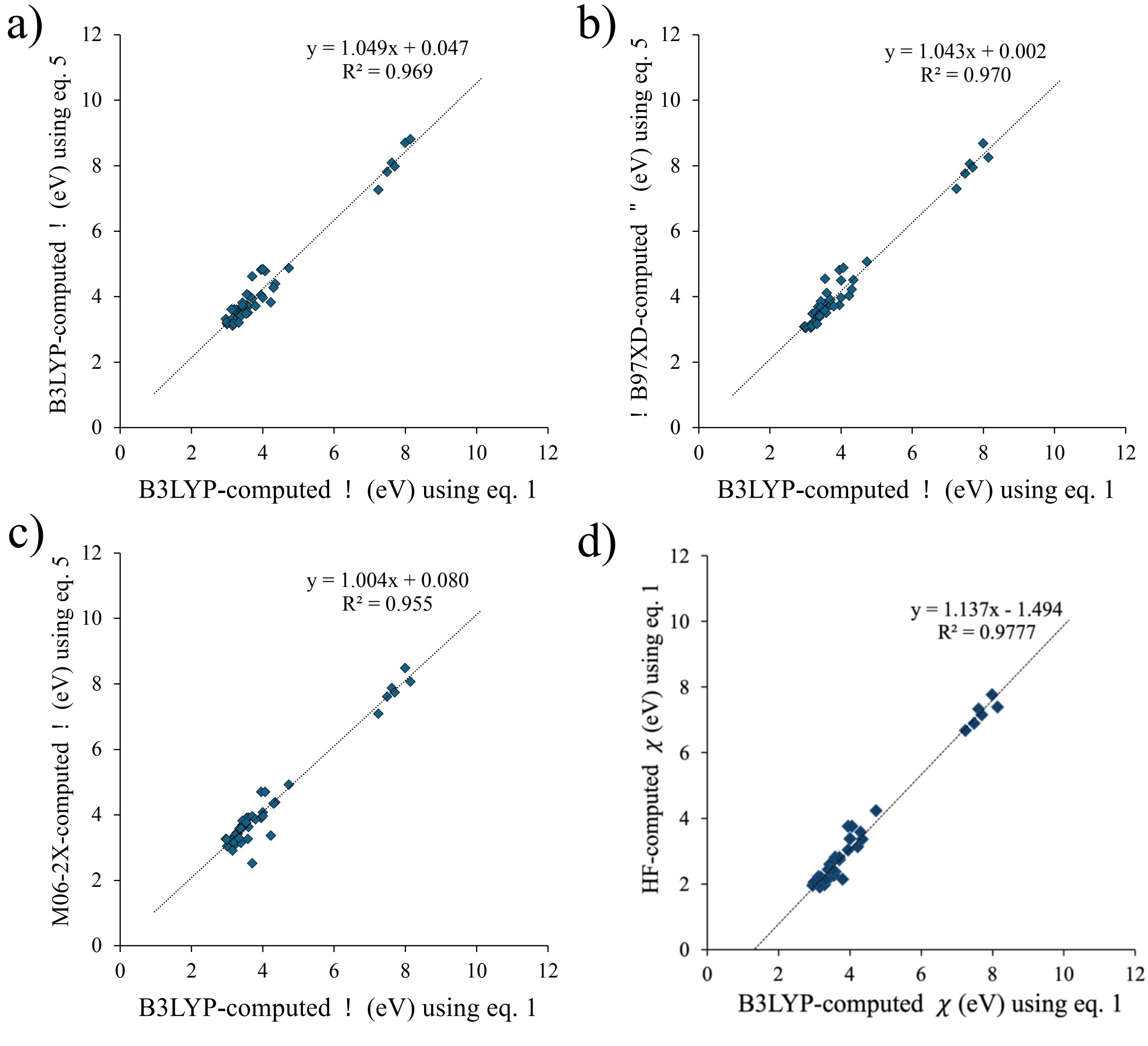

Figure S3. Correlation between B3LYP-computed hardness quantities using eq. 2 and hardness values computed using a) B3LYP (6-311++G(d,p) and eq. 6), b) ωB97XD (6-311++G(d,p) and eq. 6), c) M06-2X (6-311++G(d,p), and eq. 6), and d) HF (6-31G(d,p)) and eq. 2).

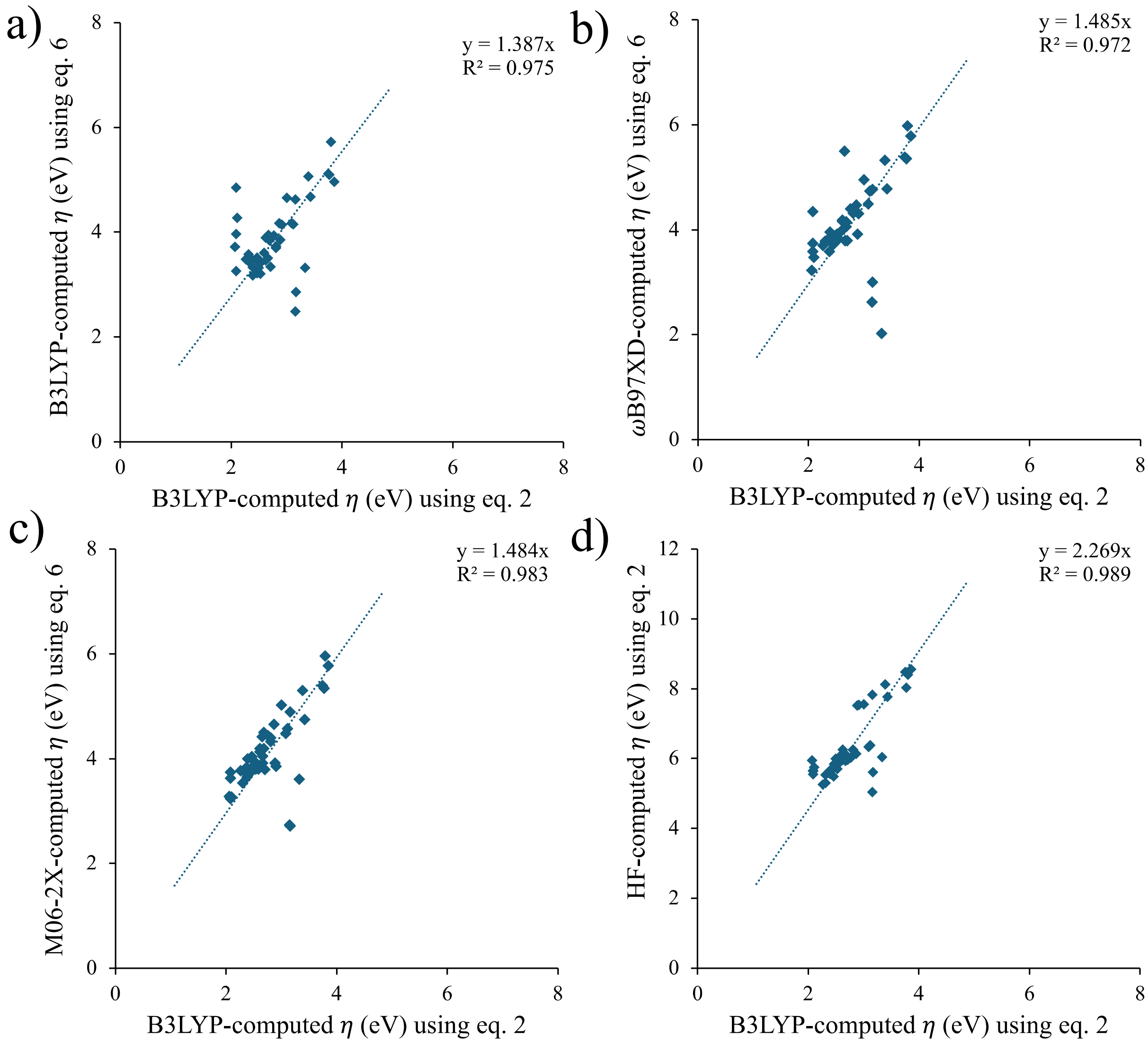

Figure S4. The residual plots of various GCN-ANN predicted energy quantities in Figure 6 for the 45 neurochemicals (28 neurotransmitters and 17 antidepressants). The standard errors (SE) in the regression analysis for HOMO, LUMO, χ, and η are 1.8, 1.1, 1.4, and 0.2 eV, respectively. Outliers for the HOMO, LUMO, and χ (shown in rectangular box), were identified when the condition residuals ≥ 1.5* SE was satisfied.

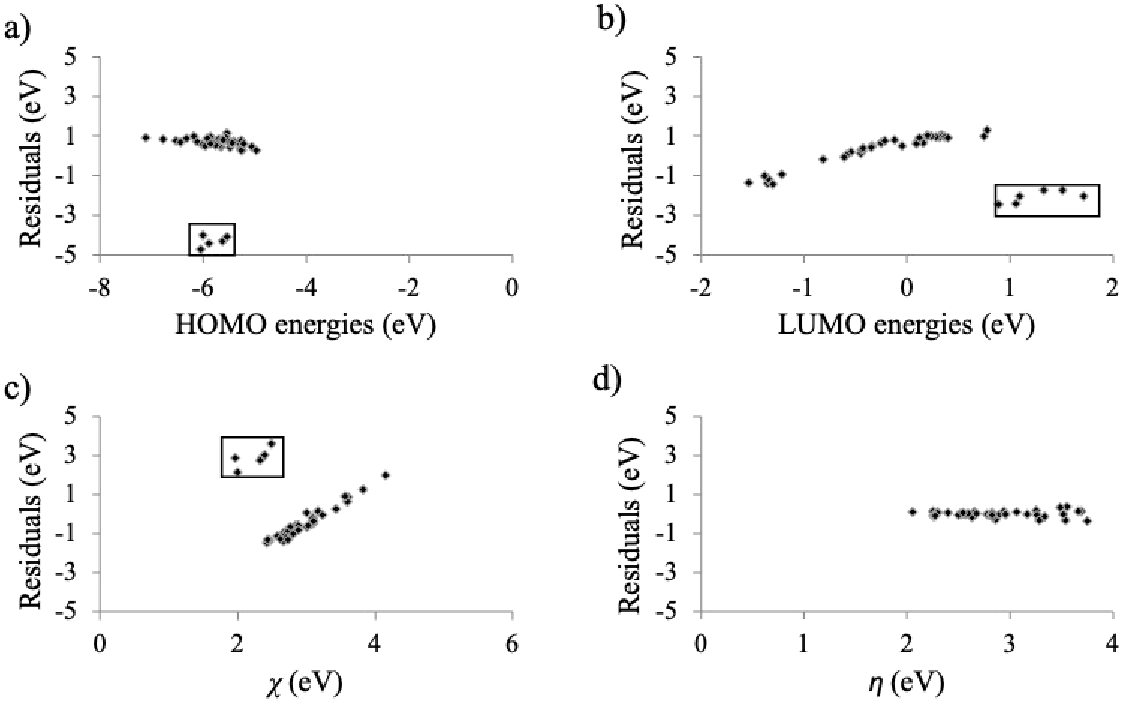

Figure S5. Trend of the binding affinity for the 28 neurotransmitters and 17 antidepressants for the $GABA_A$ receptor (PDB code: 8g5f). The positive values indicate that the active site pocket is small, and steric hindrance plays a major role. A line of best fit for only actives (i.e., molecules exhibiting negative affinity) yielded a value of 0.37.

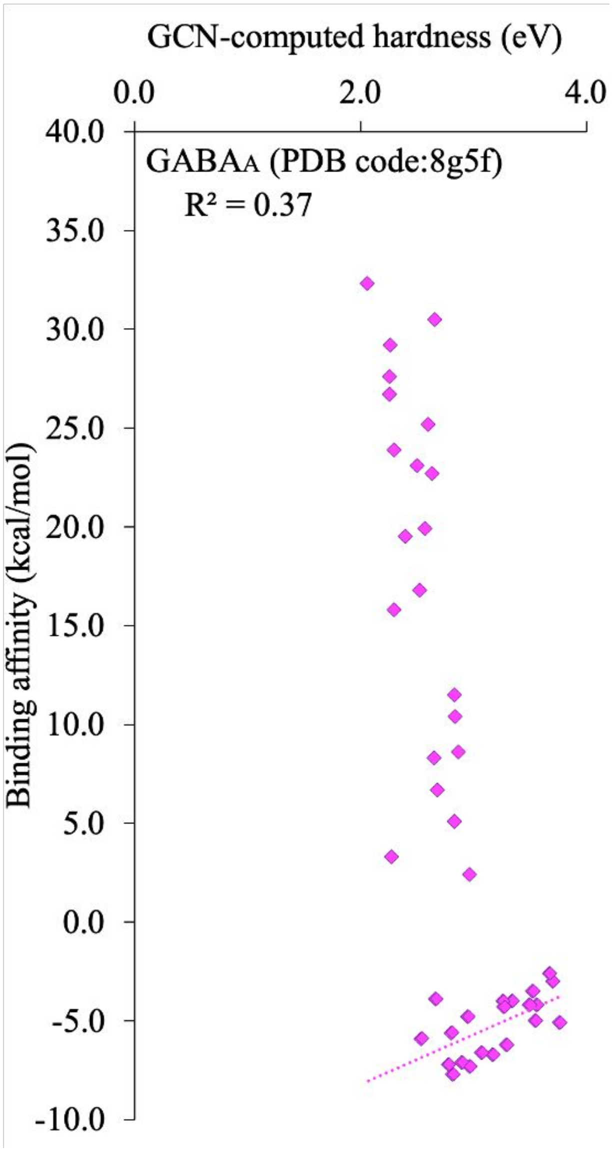

Figure S6. Common cluster of molecules of similar substructures appearing in a) 5-HT1A receptor (PDB code: 7e2y) and b) NAT (PDB code: 8wtv) as compared to the M3R receptor (PDB code: 4u14) as a reference. The numbers correspond to molecules whose chemical structures are provided in Fig. 10.

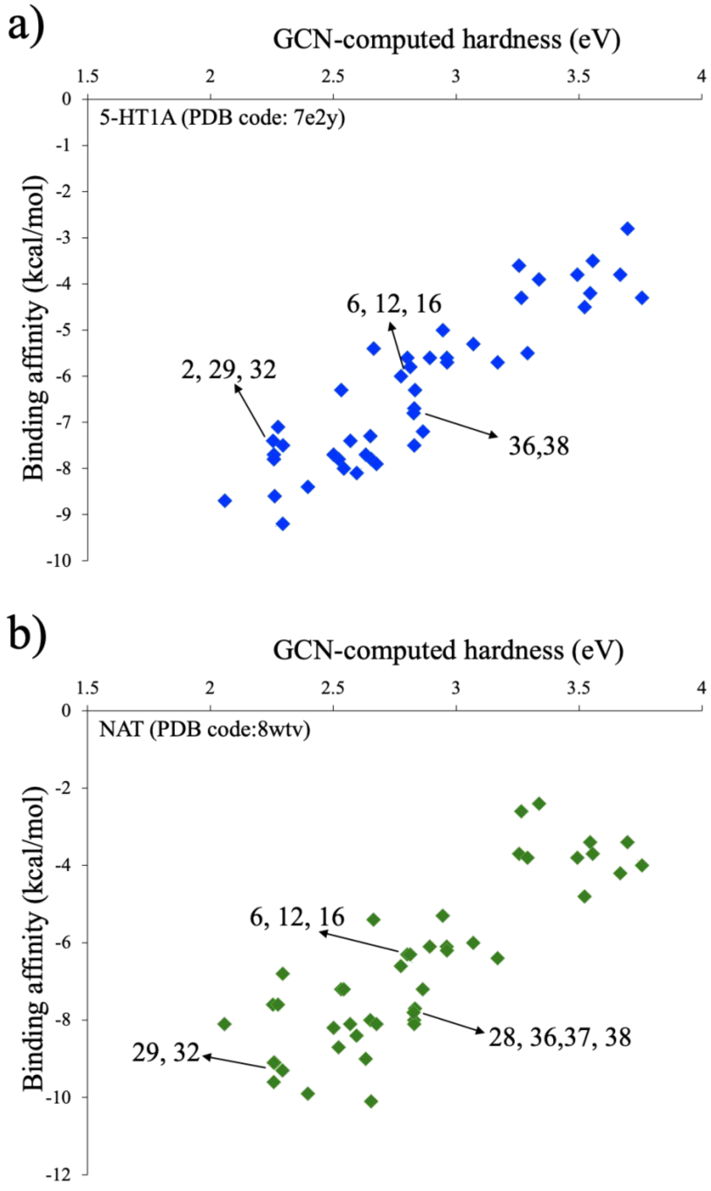

Supplement: Supplementary file 1 [file ci5c00724_si_001.pdf]
